# *C*. *elegans* ZHP-4 is required at multiple distinct steps in the formation of crossovers and their transition to segregation competent chiasmata

**DOI:** 10.1371/journal.pgen.1007776

**Published:** 2018-10-31

**Authors:** Hanh Nguyen, Sara Labella, Nicola Silva, Verena Jantsch, Monique Zetka

**Affiliations:** 1 Department of Biology, McGill University, Montreal, Quebec Canada; 2 Department of Chromosome Biology, Max F. Perutz Laboratories, Vienna Bio Center, University of Vienna, Vienna, Austria; University of Iowa, UNITED STATES

## Abstract

Correct segregation of meiotic chromosomes depends on DNA crossovers (COs) between homologs that culminate into visible physical linkages called chiasmata. COs emerge from a larger population of joint molecules (JM), the remainder of which are repaired as noncrossovers (NCOs) to restore genomic integrity. We present evidence that the RNF212-like *C*. *elegans* protein ZHP-4 cooperates with its paralog ZHP-3 to enforce crossover formation at distinct steps during meiotic prophase: in the formation of early JMs and in transition of late CO intermediates into chiasmata. ZHP-3/4 localize to the synaptonemal complex (SC) co-dependently followed by their restriction to sites of designated COs. RING domain mutants revealed a critical function for ZHP-4 in localization of both proteins to the SC and for CO formation. While recombination initiates in *zhp-4* mutants, they fail to appropriately acquire pro-crossover factors at abundant early JMs, indicating a function for ZHP-4 in an early step of the CO/NCO decision. At late pachytene stages, hypomorphic mutants exhibit significant levels of crossing over that are accompanied by defects in localization of pro-crossover RMH-1, MSH-5 and COSA-1 to designated crossover sites, and by the appearance of bivalents defective in chromosome remodelling required for segregation. These results reveal a ZHP-4 function at designated CO sites where it is required to stabilize pro-crossover factors at the late crossover intermediate, which in turn are required for the transition to a chiasma that is required for bivalent remodelling. Our study reveals an essential requirement for ZHP-4 in negotiating both the formation of COs and their ability to transition to structures capable of directing accurate chromosome segregation. We propose that ZHP-4 acts in concert with ZHP-3 to propel interhomolog JMs along the crossover pathway by stabilizing pro-CO factors that associate with early and late intermediates, thereby protecting designated crossovers as they transition into the chiasmata required for disjunction.

## Author summary

The creation of a viable individual from the fusion of egg and sperm requires that they each contain the correct number of chromosomes. This is ensured through the meiotic divisions, which initially fasten identical chromosomes through DNA linkages that hold them together until the cell is ready to separate them. To make these linkages, called crossovers, the cell breaks the DNA in many places, and must repair them to create a crossover, or a noncrossover. We investigate here the role of ZHP-4, and its partner ZHP-3 which form a complex that associates along paired chromosomes and finally with crossover sites. ZHP-3/4 are conserved proteins found in many organisms that function in recruiting proteins required to decide which DNA event will become a crossover and how this DNA event is coordinated with changes in chromosome structure. Using mutations that reduce the function of ZHP-4, we show that the complex cannot localize normally to meiotic chromosome and that crossing over fails. Our results suggest that ZHP-3/4 work at early and late steps in the process to stabilize other factors required for crossover formation.

## Introduction

During the two specialized divisions of meiosis, a single round of DNA replication is followed by two rounds of segregation that ultimately produce gametes with half the parental number of chromosomes. Central to chromosome segregation accuracy is the formation of chiasmata between paired homologous chromosomes that are the visible product of genetic crossing over. The critical series of events leading to the formation of these linkages occurs during meiotic prophase when programmed meiotic DNA double-strand breaks (DSBs) are repaired using homologous recombination (HR). An early step in this process is resection of a DSB end to form a single-stranded stretch of DNA that can recruit Rad51, an event that initiates invasion of the homologous chromosome and the formation of a joint molecule (JM) intermediate to link the homologs (reviewed in [1]). Resolution of these JMs can proceed through a crossover (CO) or noncrossover (NCO) pathway and the route chosen at any given site is carefully monitored. To ensure crossover formation, the number of induced DSBs is in excess of the final number of COs (reviewed in [2]), however, the number of crossovers is in turn strictly regulated in any given organism (*e*.*g*. [3,4]). Consequently, a decision must be made to stabilize certain JM intermediates for entry into the CO pathway, while the remaining events are repaired as NCOs [5]. These events are particularly tightly regulated in *Caenorhabditis elegans*, where an estimated 5–12 DSBs along a chromosome pair must be processed to yield a single exchange event [6–8] that serves to both physically link the homologous chromosomes and asymmetrically reconfigure the bivalent in preparation for interaction with the segregation machinery (reviewed in [9]). *C*. *elegans* meiotic chromosomes exhibit robust interference that effectively limits each homolog pair to a single crossover [10–11]. As in other organisms, CO formation in the nematode is promoted by conserved players that act to stabilize and protect JM intermediates in the CO pathway, including the scaffolding protein RMH-1 (RM1), MSH-4/5 (MutSγ), and cyclin-like COSA-1(CNTD) [11–14]. In addition to these factors, a family of proteins resembling SUMO E3-like ligases has emerged as pivotal regulators of the decision to transform JM recombination intermediates into crossovers [15–17]. The canonical budding yeast Zip3p [18] exhibits E3 SUMO activity *in vitro* [19] and orthologs have subsequently been identified in mammals, plants, nematodes, other fungi, and *Drosophila* [20–29]. Members of the Zip3 E3-ligase related family are required for CO formation and share similar protein structures: an N-terminal RING finger domain, followed by a coiled-coil domain and a C-terminal domain enriched in serine residues [25]. In these organisms, the Zip3-like proteins diverge into two possible clades, one defined by Zip3, the vertebrate RNF212 and nematode ZHP-3/ZHP-4, and the other represented by HEI10 and its orthologs [21,23,29]. Budding yeast possesses a single member of the Zip3/RNF212 group [18], while plants and the filamentous fungus *Sordaria* appear to carry a single ortholog of the HEI10 subgroup [23,24,26], and *C*. *elegans* and mammals possess members from both subgroups [20,21,25,27,29]. However, all members of both groups share a similar pattern of localization by appearing as numerous foci or stretches along the synaptonemal complex (SC) and eventually persisting at the few obligate CO sites. In *C*. *elegans*, for example, a predicted ZHP-3/ZHP-4 heterodimer is required for crossing over, localizes to the SC and finally restricts to the six late CO intermediates typically observed in each meiotic nucleus at late pachytene stages [22,29, this study]. The pattern of ZHP-3/4 localization is reminiscent of other pro-crossover factors (RMH-1, MSH-5, and COSA-1), which similarly begin with abundant early localization that is then confined to the sites of the obligate crossovers at late pachytene stages, and finally disappears as chromosomes desynapse and chiasmata emerge [11,12].

An elusive question in the study of meiosis is how the well-studied molecular events of DNA strand exchange that lead to CO formation transform into the microscopically evident chiasmata required for chromosome segregation. Early microscopy studies of these events revealed a physical connection (chiasma) between chromatids [30] and their correlation in number with the frequency of genetic exchange [31]. Consequently, while it is widely accepted that chiasmata originate with the formation of crossovers, the question of how HR at the DNA level becomes a cytologically evident chiasma capable of supporting chromosome segregation remains largely unexplored. In the case of *C*. *elegans*, the emergence of chiasmata is coupled to remodelling of the bivalent in preparation for interaction with the spindle machinery and regulated cohesion loss [32,33]. In this study, we have shown that *zhp-3/4* are required at distinct stages in this transition. First, we show that at early pachytene stages, *zhp-3/4* are required to promote the formation of an RMH-1-mediated JM competent intermediate to recruit pro-CO factors. Second, we show that *zhp-3/4* are required at late pachytene exit stages for the transition from the late crossover intermediate (likely the double Holliday Junction, dHJ [34]) to chiasmata. In fact, *zhp-4* is required to stabilize RMH-1 at early JMs and is necessary for recruitment/stabilization of MSH-5 and signalling the end to meiotic DSB induction. Furthermore, genetic crossovers that occur in *zhp-4* mutants and are not marked by the pro-crossover factors (RMH-1, MSH-5, COSA-1) are unable to form chiasmata capable of triggering the bivalent remodelling required for accurate chromosome segregation at meiosis I. Together, our data suggest that the ZHP-3/4 complex is recruited to the SC as it forms [22] to convene the complex in proximity to early recombination intermediates where it stabilizes pro-crossover factors that first promote JM resolution along the crossover pathway and finally resolve crossover-designated sites into chiasmata.

## Results

### *zhp-4* encodes a ZHP-3 paralog that is required for crossover formation and ZHP-3 localization

**Fig 1:**
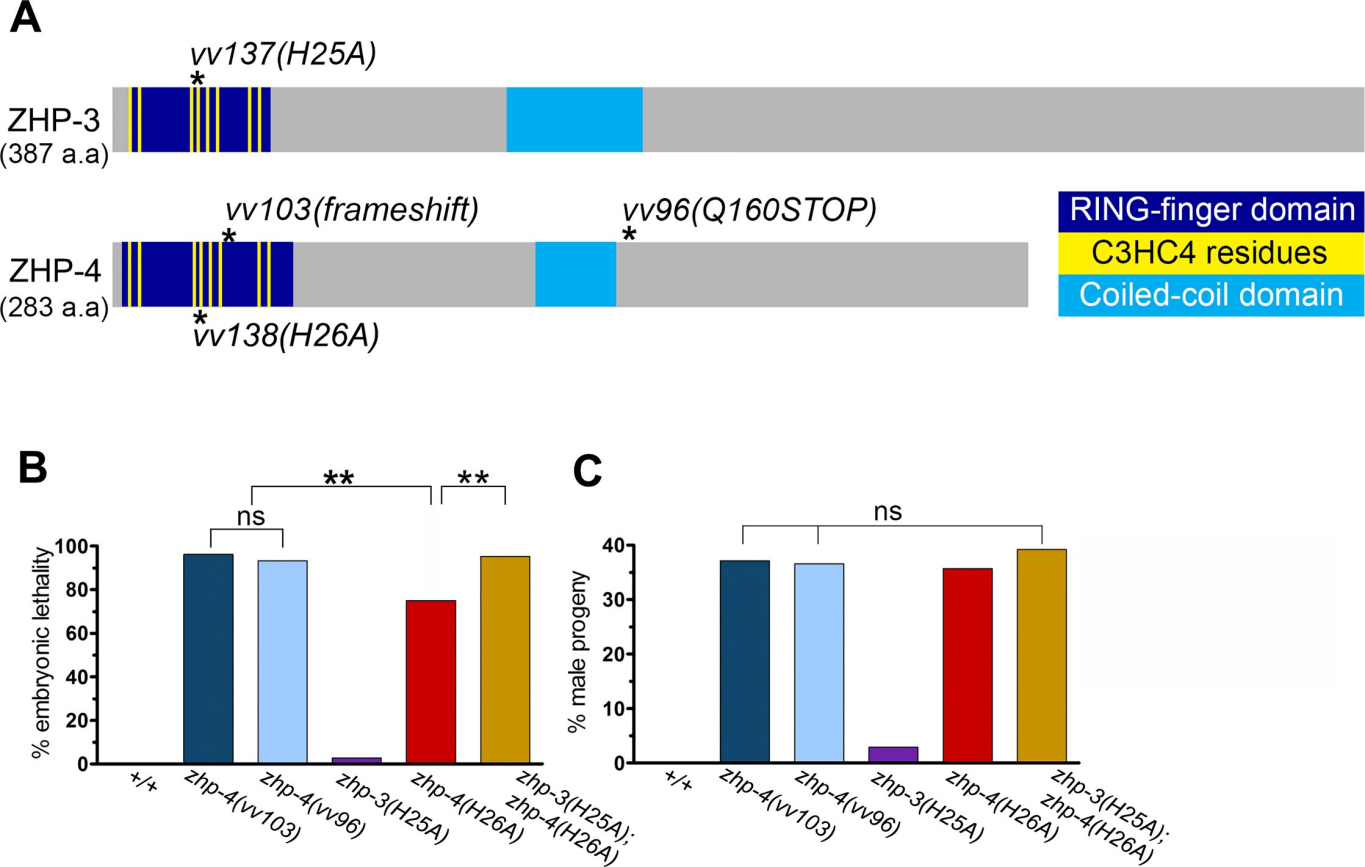
*zhp-4* and *zhp-3* are paralogs that share a conserved SUMO E3 ligase-type structure and are required for chromosome segregation. (A) Schematic of the predicted protein structures of ZHP-3 and ZHP-4: RING finger domain (dark blue), eight conserved Zn^2+^-coordinating residues (yellow) and coiled-coil domain (light blue) and location of all mutations used in this study (asterisks). All protein domains were predicted using InterPro (https://www.ebi.ac.uk/interpro/). *zhp-4(vv103)* is defined by a frameshift mutation at a.a. 36 that results in a premature stop codon at position a.a. 56. *zhp-4(vv96)* results in a premature stop codon at a.a. 160, leading to deletion of the C-terminus of the protein. The *zhp-3(vv137[H25A])* and *zhp-4(vv138[H26A])* alleles are predicted to disrupt the folding of the RING finger domains of ZHP-3 and ZHP-4. (B) Quantification of embryonic lethality and (C) frequencies of male offspring among the progeny of the indicated genotypes. P_o_ individuals scored: *+/+* n = 10, *zhp-4(vv103)* n = 30, *zhp-4(vv96)* n = 13, *zhp-3(H25A)* n = 10, *zhp-4(H26A)* n = 10, *zhp-3(H25A);zhp-4(H26A)* n = 15. Embryonic lethality (Emb) and High incidence of males (Him) values for individual broods were initially transformed with arc-cosine function, and assessed by ANOVA followed by multiple pairwise coparison tests (ns = not significant and ** *p*<0.01).

An EMS screen for recessive nondisjunction mutants (Materials and Methods) isolated a mutation (*vv96)* in Y39B6A.16 (ZHP-4), a gene with significant predicted protein sequence similarity to ZHP-3 (13% identity and 23% similarity). Since ZHP-4 and ZHP-3 share the structural features of the C_3_HC_4_-type RING finger domain characteristic of known SUMO E3 ligases (Fig 1A; reviewed by [35]), we investigated its role in meiosis and its relationship to ZHP-3.

The *zhp-4(vv96)* mutation results in a premature translation termination codon at amino acid (a.a.) 160 that is predicted to produce a truncated protein with an intact RING finger domain (Fig 1A) and is compatible with other data presented below that it represents a severely hypomorphic allele. To determine the null phenotype, CRISPR-Cas9 mutagenesis (Materials and Methods) was used to generate the deletion allele *zhp-4(vv103)*, a frameshift mutation that creates a premature stop codon before the last two cysteines in the predicted RING-finger domain (a.a. 56; Fig 1A). The germlines of both *zhp-4* mutants displayed no overt defects in nuclear morphology as assessed by DAPI staining (S1A Fig). The broods of *zhp-4(vv96)* and *zhp-4(vv103)* mutant homozygotes were marked by statistically similar levels of high embryonic lethality and incidence of XO males amongst the surviving progeny, phenotypic hallmarks of autosomal and X-chromosome nondisjunction ([36] Fig 1B and 1C).

**Fig 2:**
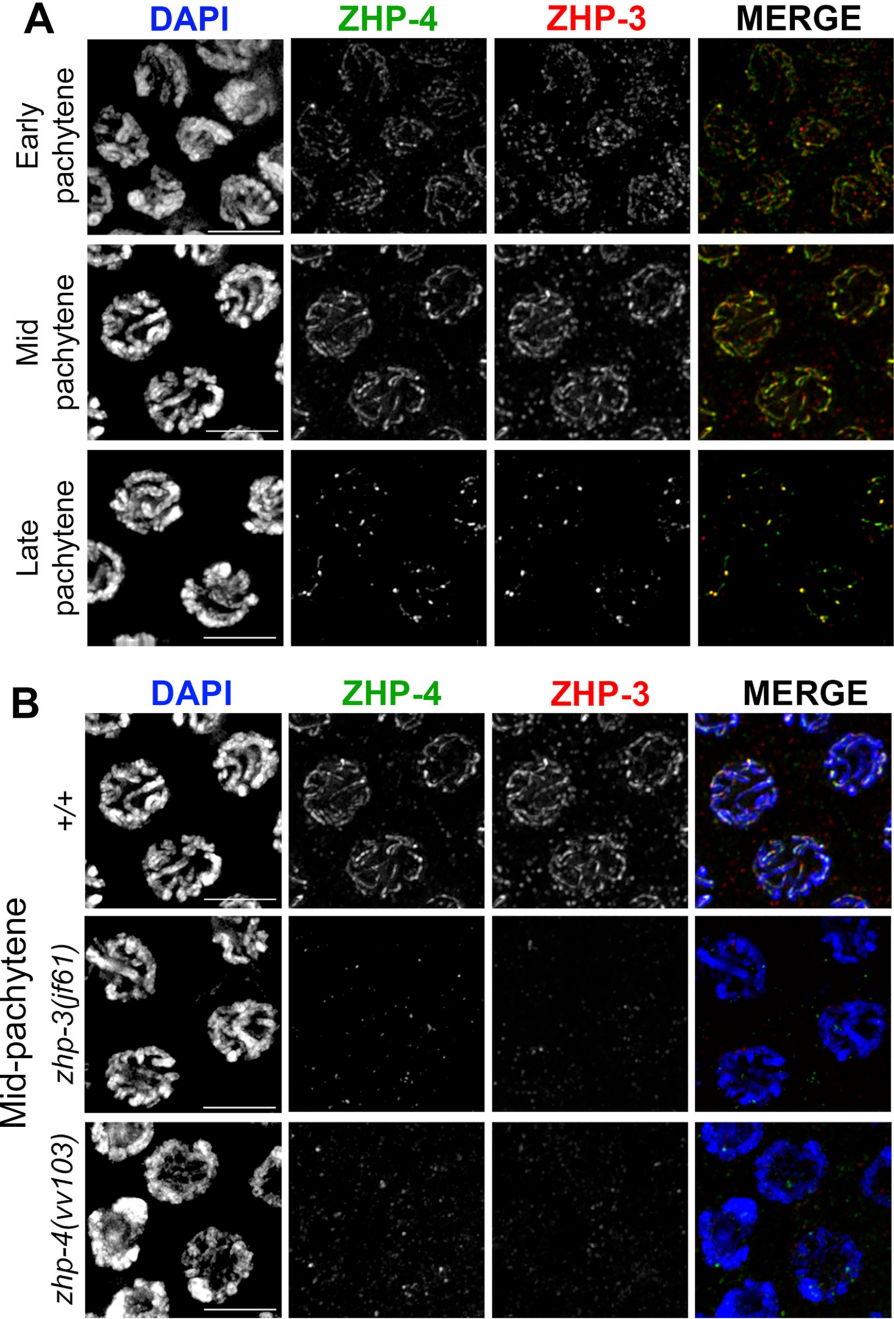
ZHP-4 and ZHP-3 colocalize and are interdependent for their localization to the SC and crossover sites. (A) α-ZHP-4 (green) and α-ZHP-3 (red) immunostaining in wild-type pachytene stage nuclei showing colocalization with the SC in early and mid-pachytene followed by co-restriction to ~6 distinct foci per nucleus in late pachytene. (B) Immunostainings of mid pachytene region nuclei of the indicated genotypes with α-ZHP-4 (green) and α-ZHP-3 (red). ZHP-4 could not be detected above background levels in *zhp-3(jf61)* germlines stained with DAPI (blue) and conversely no ZHP-3 could be detected in *zhp-4(vv103)* mutants. Scale bars 5 μm.

Previous studies observed that ZHP-3 first localizes to synapsed chromosomes in an SC-dependent manner, and is then restricted at late pachytene stages to foci that correspond to sites of crossing over [21,22]. To investigate if ZHP-4 functions with its paralog ZHP-3 in CO formation, we first examined the localization of the proteins during meiotic prophase and tested their codependency in recruitment to chromosomes (Fig 2). In the case of ZHP-4, antibodies raised against the C-terminal 123 a.a. and a CRISPR-generated HA tag (Materials and Methods) both revealed that ZHP-4 localizes to the SC from earliest pachytene and is similarly restricted to the 5–7 late pachytene foci reported for ZHP-3 (Figs 2A and S2A; [21,22]). Like ZHP-3, ZHP-4 recruitment to chromosomes is SC dependent (S2C Fig), and the protein colocalizes with ZHP-3 at the SC and at the CO sites that emerge at late pachytene (Fig 2A). ZHP-3 localization was reduced to weak background levels throughout meiotic prophase in the absence of ZHP-4 (Fig 2B), and conversely ZHP-4 localization was similarly abrogated in *zhp-3(jf61)* mutant germlines (Fig 2B), indicating that the two paralogs are co-dependent in their recruitment to meiotic chromosomes. Our observations that ZHP-3/4 colocalize in a co-dependent manner to the same chromosome features and the results of a recent characterization of the ZHP-3 family [29] are most simply reconciled with a model in which the two paralogs physically cooperate to mediate the formation of crossovers.

**Fig 3:**
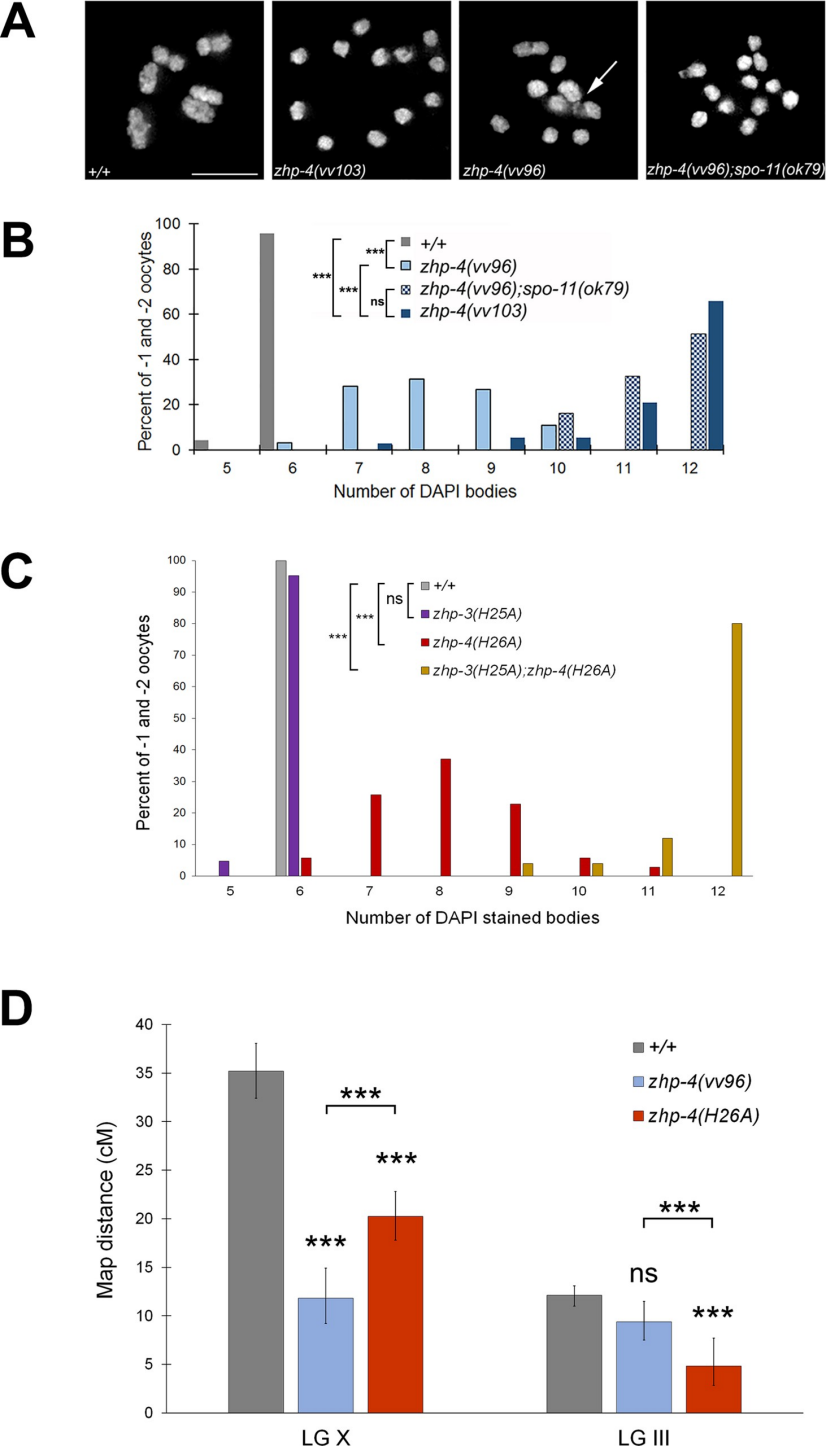
*zhp-4* mutants are compromised for crossing over. (A) Representative full projections of diakinesis nuclei of the indicated genotypes stained with DAPI. In comparison to the six bivalents observed in wild types, *zhp-4(vv103)* mutants contain no bivalents, indicating a loss of crossover formation. In contrast, *zhp-4(vv96)* mutants nuclei contain a mixture of structures, including univalents, anomalous bivalents ‘tethered’ by chromatin (white arrow), and bivalents. *zhp-4(vv96);spo-11(ok79)* nuclei contain mostly univalents, indicating that the bivalents displayed by *zhp-4(vv96)* are recombination initiation dependent. (B) Histogram showing distribution of the number of DAPI bodies in diakinesis nuclei in the -1 and -2 oocytes for the indicated genotypes. Nuclei scored: *+/+* n = 97, *zhp-4(vv103)* n = 38, *zhp-4(vv96)* n = 64, *zhp-4(vv96);spo-11(ok79)* n = 30. Using Kruskal-Wallis and Dunn’s post test, all pairwise comparison results are significantly different (*** *p*<0.001) except for the comparison between *zhp-4(vv103)* and *zhp-4(vv96);spo-11(ok79)* nuclei (ns *p*>0.05). (C) Bar graph showing quantification of the numbers of DAPI-stained bodies in diakinesis nuclei at the -1 and -2 positions in germlines from animals of the indicated genotype. Most *zhp-3(H25A)* nuclei have 6 DAPI bodies and are not significantly different from wild types while the increase observed in *zhp-4(H26A)* mutants is significantly different from both. *zhp-3(H25A);zhp-4(H26A)* has on average 12 DAPI bodies indicating a severe loss of function (significantly different from all other genotypes assessed). Statistical significance assessed by Kruskal-Wallis test and post Dunn’s test: ns = not significant, *p*>0.05, *** *p*<0.001. Nuclei scored: *+/+* n = 20, *zhp-3(H25A)* n = 21, *zhp-4(H26A)* n = 35, *zhp-3(H25A);zhp-4(H26A)* n = 25. (D) Bar graph showing the frequency of genetic recombination in two large genetic intervals in wild types and *zhp-4* mutants (detailed in Materials and Methods). Multiple pairwise coparisons indicate that wild types and *zhp-4* mutants are all significantly different from one another in the X chromosome *dpy-3(e27) unc-3(e151)* interval. In the *dpy-18(e364) unc-25(e156)* interval on chromosome III, no statistical difference was observed between wild types and *zhp-4(vv96)* mutants, but *zhp-4(H26A)* mutants are statistically different from both wild types and *zhp-4(vv96)* mutants. Data are represented as recombination frequency results ± 95% confidence interval and statistical analysis was conducted using Chi-squared test and Bonferroni corrections on the raw count of phenotypes (ns *p*>0.05, *** *p<*0.001).

Consistent with the crossover-specific function of ZHP-3 [21], ZHP-4 is similarly not required for pairing, synapsis (S1B and S1C Fig), or for meiotic DSB induction [29] as evidenced by the formation of foci of the strand exchange RAD-51 protein [37,38]. While wild-type diakinesis oocytes stained with DAPI invariably contained 6 bivalents (representing the 12 chromosomes linked by chiasmata), *zhp-4(vv103)* and *zhp-4(vv96)* mutants respectively exhibited an average of 11.4 and 8.2 DAPI-stained figures (*p* < 0.001 compared to WT, Fig 3A and 3B), instead of the 12 predicted in the event of complete loss of crossover potential [39]. To directly assess the effect of loss of ZHP-4 function on crossing over, we genetically measured the frequency of genetic exchange between visible markers (Fig 3D; Materials and Methods) in large genetic intervals comprising ~ 1/3 of the chromosome III (12 m.u.) and ~3/4 of the X chromosome (38 m.u.). We were unable to measure crossing over in *zhp-4(vv103)* null mutants, as the mutation in combination with several visible markers tested was near inviable (could not be maintained as a strain) and the embryonic lethality and aneuploidy phenotypes made it impossible to attain data sets for statistical comparisons; however, rare recombinants (<1/100 wild-type progeny) that segregated progeny of the recombinant phenotype were recovered in both intervals. The frequency of isolation of the rare recombinants in *vv103* mutants is similar to the rare events previously reported for *zhp-3* null mutants (2/93) [21]. In contrast, *zhp-4(vv96)* mutants attained 33% of wild-type crossover levels on the X chromosome (*p* < 0.001) and 77% of wild-type crossover levels on chromosome III (*p* > 0.05), indicating that *vv96* mutants remained competent for significant levels of genetic exchange.

### The RING finger domain of ZHP-4 is required for ZHP-3/ZHP-4 SC localization and wild-type levels of chiasma formation

ZHP-3/4 contain a conserved C_3_HC_4_-type RING finger domain required for the catalytic activities of E3 ubiquitin and SUMO ligases (reviewed by [35,40]). We investigated its contribution to ZHP-3/ZHP-4 function by targeting conserved histidine residues at positions known to be essential for RING finger function during meiosis in other organisms (S3 Fig); for example, *S*. *cerevisiae zip3*^*H74A*^ *and zip3*^*H80A*^ mutants exhibit defects in SC assembly and sporulation efficiency [19], while *Sordaria hei10*^*H30A*^ mutants are defective in crossing over and chiasma formation [26]. While *zhp-4(H26A)* mutants exhibited high embryonic lethality and males amongst surviving progeny (*p* < 0.01 compared to WT), *zhp-3(H25A)* mutants produced only 3% dead embryos and 3% male progeny (*p* > 0.05 in comparison to WT, Fig 1B and 1C), indicating that the RING domain of ZHP-3 is largely expendable for its function. The severity of the embryonic lethality defects observed in the two mutants was also reflected at diakinesis where only 5% of *zhp-4(H26A)* nuclei exhibited the 6 DAPI bodies observed in wild-type nuclei (average of 8.0, *p* < 0.001), while 95% of *zhp-3(H25A)* diakinesis nuclei (average of 5.9, *p* > 0.05) did so (Fig 3C). Since neither *zhp-3(H25A)* nor *zhp-4(H26A)* single mutants replicated the phenotypes of the respective null mutant, we addressed the possibility of RING domain redundancy by examining the consequence of loss of both RING domains. *zhp-3(H25A); zhp-4(H26A)* double mutants exhibited phenotypes that were more severe than those of *zhp-4(H26A)* single mutants and not different from those observed for the null mutant; homozygotes segregated 95% dead embryos and 43% male progeny (Fig 1B and 1C, *p* > 0.05) and 80% of diakinesis nuclei showed 12 univalents (average of 11.7 *p* < 0.001; Fig 3C).

**Fig 4:**
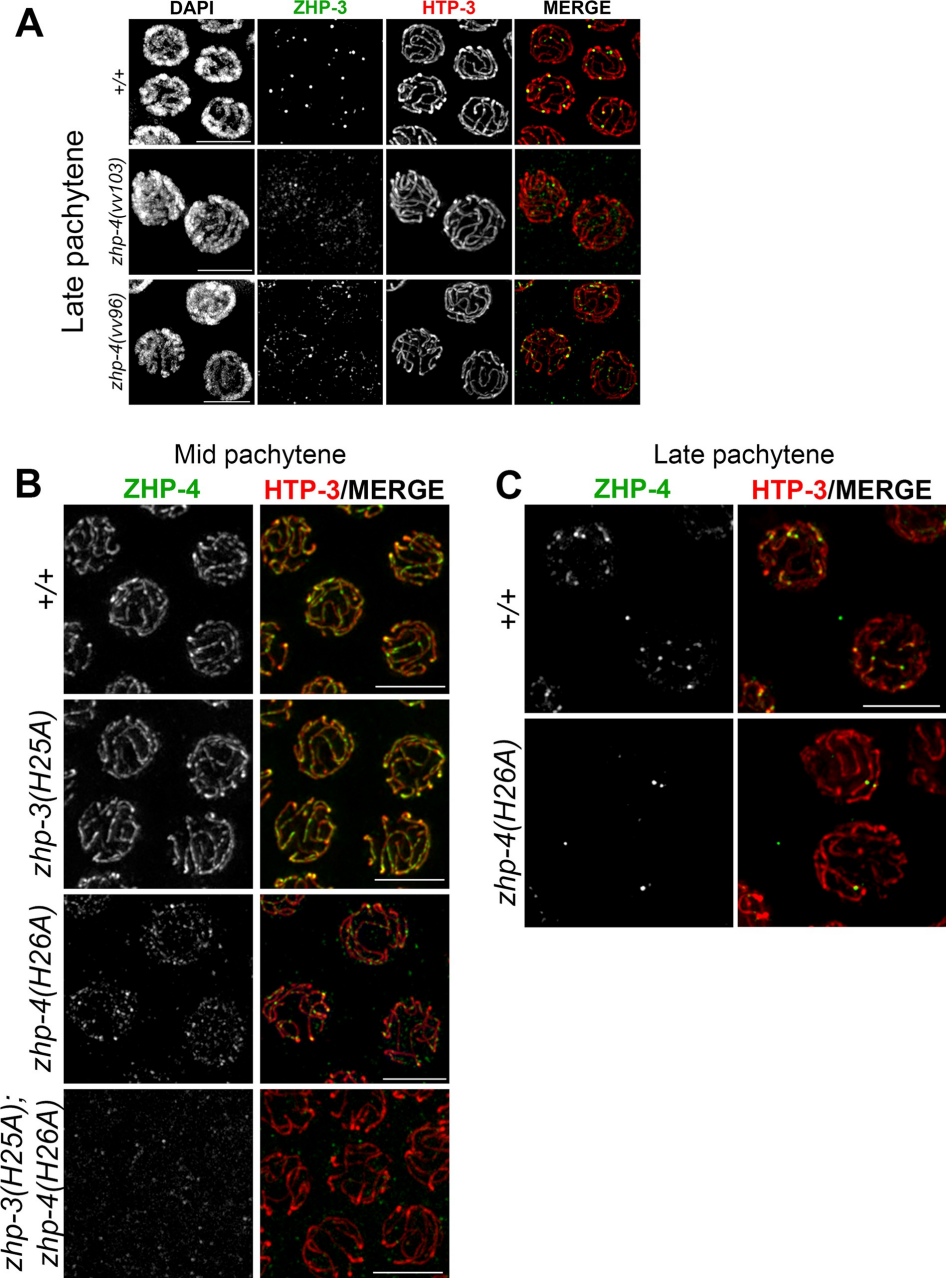
The RING finger domain of ZHP-4 is required for localization of ZHP-3/4. (A) Representative images of late pachytene nuclei of wild types, *zhp-4(vv103)* and *zhp-4(vv96)* mutants stained with α-ZHP-3 (green) and α-HTP-3 (red). In wild-type, ZHP-3 restricted to ~6 foci designating crossover sites per nucleus, while in *zhp-4(vv103)* mutants, no ZHP-3 SC localization or foci could be detected). In *zhp-4(vv96)* mutants, ZHP-3 foci and occasionally small stretches were detected and colocalized with synapsed HTP-3 marked axes. (B) Representative images of mid-pachytene nuclei of the indicated genotypes stained for α-ZHP-4 (green) and α-HTP-3 (red). Both wild-type and *zhp-3(H25A)* mutant nuclei show ZHP-4 localization with synapsed chromosome axes. In *zhp-4(H26A)* mutant nuclei only a punctate staining of ZHP-4 is detectable; occasional small brighter foci that colocalize with HTP-3 amongst a larger population of weak dimmer foci distributed throughout the nucleus. In *zhp-3(H25A);zhp-4(H26A)* double mutants ZHP-4 is not detectable above background levels. (C) Representative images of late pachytene nuclei stained with α-ZHP-4 (green) and α-HTP-3 (red). Wild-type nuclei largely display ~ 6 ZHP-4 foci on chromosome axes and 1–3 similarly-sized bright foci can be detected in *zhp-4(H26A)* RING mutants. Scale bars 5 μm.

In *zhp-3(H25A); zhp-4(H26A)* double mutants, ZHP-4 was not detectably recruited to synapsed chromosomes at any stage, and no enriched nuclear localization could be detected (Fig 4B). We could not detect ZHP-3^*H25A*^ localization in the double RING mutant using α-ZHP-3 antibodies (the aliquot did not provide a reliable signal even in wild-type controls); however, its localization is likely to be equally abrogated given the co-dependent co-localization of the proteins and the relative fertility of *zhp-3(H25A)*. In the case of *zhp-3(H25A)* single mutants, ZHP-4 adopted the wild-type pattern of SC localization throughout pachytene (Fig 4B) to finally restrict to ~ 6 foci marking putative crossover/chiasma sites that correlate with the lack of meiotic defects in this mutant. In *zhp-4(H26A)* single mutants, however, ZHP-4^*H26A*^ localization to the SC was reduced, discontinuous, and evident only as punctate foci of varying intensities (Fig 4B), indicating that an intact ZHP-4 RING domain is required for the contiguous pachytene pattern of ZHP-3/4 association with the SC that is observed in wild-type. Despite this disrupted localization during early/mid-pachytene stages, 1–3 bright ZHP-4^*H26A*^ foci/nucleus emerged at late pachytene (Fig 4C); this in combination with the detection of significant levels genetic crossing over (57% and 40% of wild-type chromosome X and III frequencies; Fig 3C) and the presence of bivalents at diakinesis (Fig 3C) indicates that *zhp-4(H26A)* mutants can support reduced levels of crossover formation and localization of the protein to those sites. Our results are collectively consistent with a model in which 1) the RING domain of ZHP-4 is critical for the localization of the heterodimer to the SC where it cooperates with ZHP-3 to promote crossover formation and 2) the ZHP-3 RING domain can partially compensate for loss of ZHP-4 RING domain activity to foster the formation of a severely reduced number of crossovers/chiasmata.

### *zhp-4* mutants exhibit elevated levels of RAD-51-marked early recombination intermediates

**Fig 5:**
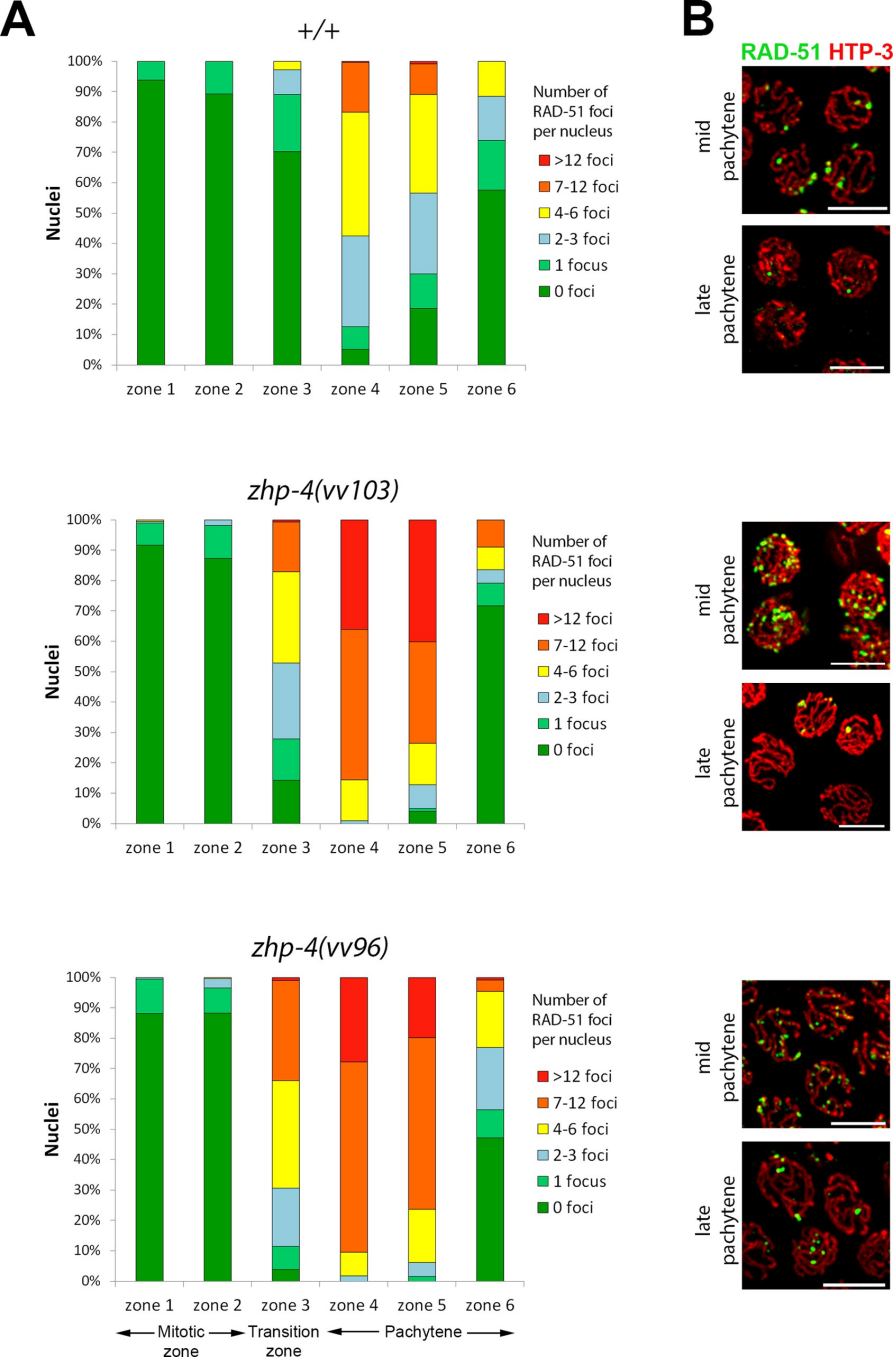
*zhp-4* mutants show elevated levels of the homologous recombination protein RAD-51 throughout prophase. (A) The number of RAD-51 foci were scored in each nucleus of a gonad from individuals of the indicated genotype, from the distal tip cells until the end of pachytene. Each gonad was then divided into six equivalent zones that are largely populated by nuclei in the following stages: mitotic region (zones 1 and 2), transition zone-early pachytene region (zone 3) and mid through-late pachytene regions (zones 4–6). As in wild-type, RAD-51 foci in *zhp-4* mutants first appear in zone 3, but at higher levels that remain statistically higher until zone 5 (*p*<0.001). At very late pachytene (zone 6), these elevated RAD-51 foci in the null allele *vv103* are reduced to wild-type levels (ns, *p*>0.05), whereas *vv96* mutants retain elevated numbers of RAD-51-marked recombination intermediates (*p*<0.001). Three gonads were analyzed for each genotype (Mann-Whitney test, *** *p*<0.001). (B) Immunolocalization of RAD-51 (green) and axis component HTP-3 (red) in representative nuclei from mid-pachytene (zone 4) and late pachytene (zone 6) stages from germlines corresponding to the genotypes in (A). Scale bars 5 μm.

In crossover-defective mutant backgrounds, RAD-51-marked recombination intermediates typically appear on time and at wild-type levels in early pachytene, however, they accumulate and persist into late pachytene stages [38]. We measured RAD-51 foci in nuclei of the mitotic (zone 1 and 2), leptotene/zygotene (referred to as the transition zone)/pachytene entry (zone 3), early pachytene (zone 4), mid-pachytene (zone 5), and late pachytene stages (zone 6) (Fig 5). In both *zhp-4(vv103)* and *zhp-4(vv96)* mutants, RAD-51 foci appeared, peaked in number, and disappeared with wild-type like timing as previously reported for *zhp-3* mutants [21], suggesting appropriate recombination initiation and timely DSB processing and repair. However, the levels of RAD-51 foci in both *zhp-4* mutants were dramatically elevated at every stage until their disappearance at late pachytene (Fig 5). In particular, meiotic RAD-51 foci first emerge in zone 3 where 70% of the wild-type nuclei have no foci (30% have 1–6 foci) while only 4% of *vv96* and 14% of *vv103* mutant nuclei lack any foci; at this same stage, 34% of *vv96* and 17% of *vv103* mutant nuclei have >7 RAD-51 foci, a category that does not appear in wild-type germlines until zone 4. This initial increase of RAD-51 foci in *zhp-4* mutants persists into later stages, but with wild-type-like dynamics; their average number per nucleus peaks in zone 4 (4.2 in wild-type, 10.5 and 11.1 in *vv96* and *vv103* mutants, respectively. *p* < 0.001), and appropriately disappears in the last zone (1.0 in wild-type, 2.0 and 1.4 in *vv96* and *vv103* mutants, respectively. *p* < 0.01 for *vv96* and *p* > 0.05 for *vv103*). While the elevated numbers of RAD-51 foci observed in *zhp-4* mutants could originate in their impaired turnover, RAD-51 foci kinetics followed the wild-type pattern and did not exhibit the accumulation observed in other CO-defective mutants [38]. This suggests that the initial elevation that appears in concert with meiotic DSB formation is not a reflection of an impairment in processing RAD-51-marked early recombination intermediates but reflects a requirement for ZHP-4 in down regulating DSB formation. To test this possibility, we examined the levels of RAD-51 foci in a *rad-54(RNAi)* background where DSB repair is blocked and RAD-51 foci are not removed, thereby permitting quantitation of the total number of DSBs formed [41]. Hermaphrodites injected with *rad-54* RNA did not show the sterility previously reported for *rad-54* deletion mutants or *rad-54(RNAi)* [41]. However, cytological analysis at 2 days post-injection (3 days post L4 stage) revealed an increase in RAD-51 foci until early/mid-pachytene stages (zone 4) at levels comparable with those previously reported [41]. We observed that the levels of RAD-51 foci decreased in mid-pachytene (zone 5) and disappeared in late pachytene stages (zone 6), in contrast to *rad-54* mutants in which the levels remain high until the end of pachytene. This suggests a partial effect of the RNAi treatment, and consequently we measured RAD-51 foci levels up to zone 4 in *zhp-4(vv103); rad-54(RNAi)* germlines and compared them to the levels observed in the same zone in *rad-54(RNAi)* controls (S4 Fig). We observed a significant increase of RAD-51 foci in zone 4 in the double mutant in comparison to *rad-54(RNAi)* alone (average of 13.5 versus 9.6 for *rad-54(RNAi)*, *p* < 0.00001). This result is consistent with the interpretation that the higher levels of RAD-51 foci observed in *vv103* germlines are the consequence of increased DSB formation rather than defective DSB repair. This suggests a role for ZHP-4 in negatively regulating recombination initiation, possibly by being required for the formation of a crossover intermediate that can feedback to negatively regulate DSB formation (reviewed in [42]).

### ZHP-4 is required for RMH-1-marked recombination intermediates

**Fig 6:**
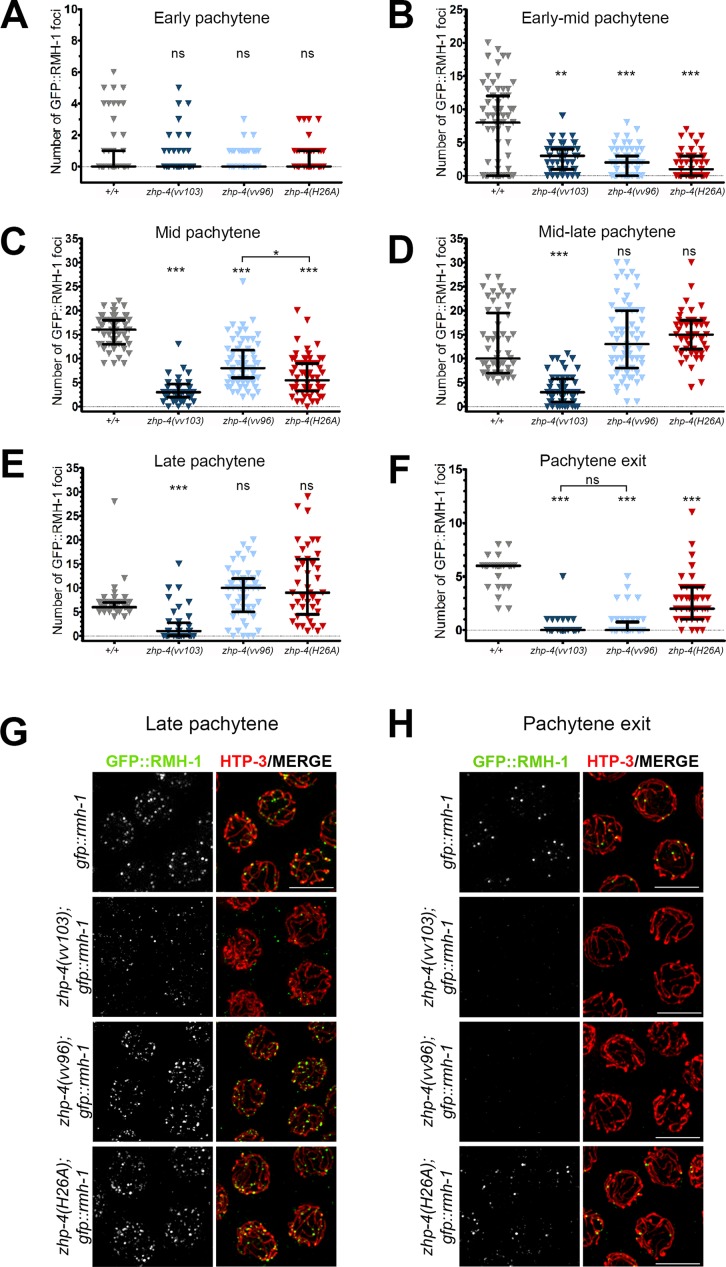
ZHP-4 is required to stabilize RMH-1 at early and late recombination intermediates. GFP::RMH-1 was quantified in the germline nuclei of animals of the indicated genotypes and the numbers binned into six equal zones encompassing the pachytene region: early (A), early-mid (B), mid (C), mid-late (D), late pachytene (E) and pachytene exit (F). All nuclear foci were quantitated. In wild types, RMH-1 appearance and kinetics are similar to a previous report [12]. Three gonads were scored for each genotype and the data are represented as individual points grouped by genotype and plotted on each Y axis; black bars represent the median +/- interquartile range and Kruskal-Wallis tests and multiple pairwise comparisons were used to assess significant differences among mutants versus wild types (ns = not significant, ** *p*<0.01, *** *p*<0.001). (G,H) Immunolocalization of GFP::RMH-1 (green) and HTP-3 (red) in late pachytene nuclei of the indicated genotypes detects a few small foci in *zhp-4(vv103)* mutants and multiple brighter foci in *zhp-4(vv96)* and *zhp-4(H26A)* mutant nuclei. At pachytene exit, no late RMH-1 foci were detectable in *zhp-4(vv103)* and *zhp-4(vv96)* mutants, while 2–4 foci with sizes similar to wild-type foci could be detected on HTP-3-marked tracks in the RING mutant. Scale bars 5 μm.

Since *zhp-4* mutants robustly initiated recombination but failed to resolve these events into crossovers, we used known markers of crossing over to probe the origin of this defect. We first characterized the dynamics of RMH-1, a conserved scaffolding component thought to co-operate with Bloom’s helicase (BLM; nematode HIM-6) during an early step in crossover designation [43] and the resolution of recombination intermediates into crossovers or noncrossovers [12]. In *C*. *elegans*, GFP::RMH-1 is first recruited to synapsed chromosomes at early pachytene in numbers exceeding the number of obligate COs, suggesting that it marks both COs and non COs at this stage; by late pachytene, the number of RMH-1 foci decreased to ~6 per nucleus putatively marking the obligate crossover sites [12]. We observed a similar kinetics of appearance and disappearance of RMH-1 foci in wild-type germlines (Fig 6A–6F) as previously reported [12]. Both *vv103* null mutants and *vv96* hypomorphs acquired low levels of RMH-1 foci in very early pachytene stages in numbers not significantly different from wild-type (*p* > 0.05, Fig 6A), indicating that ZHP-4 mutants are competent for formation of these early RMH-1-marked recombination intermediates with appropriate timing and levels. In *zhp-4(vv103)* mutants, the number of RMH-1 foci did not significantly increase throughout pachytene (*p* < 0.001), and the 2–3 foci that did form disappeared at the approach of pachytene exit (Fig 6E and 6F), similar to the timing and kinetics observed for RMH-1 foci in *zhp-3(jf61)* mutants [12].

In contrast to *vv103* null mutants, hypomorphic *zhp-4(vv96)* mutants steadily accumulated RMH-1 foci as pachytene progressed, reaching levels not different than those observed in wild types at mid-late pachytene stages (median of 10, *p* > 0.05; Fig 6D, 6E and 6G). However, the accumulation of these foci was delayed with respect to the kinetics observed in wild types germlines (Fig 6B and 6C); for example, while the number of RMH-1 foci in wild-type peaked at mid-pachytene with a median of 16 RMH-1 foci, *vv96* mutants exhibited 8 foci and displayed a peak later at mid-late pachytene with 13 foci (Fig 6D). Although *zhp-4(vv96)* mutants appeared competent in the formation of early RMH-1 marked recombination, they proved to be defective in presenting the bright RMH-1 foci at very late pachytene stages in which RMH-1 marks the sites of the obligate crossovers (Fig 6F and 6H). While in wild types the excess early RMH-1 foci disappeared to a final median population of 6, *zhp-4(vv96)* mutant nuclei exhibited no detectable foci at the stage approaching pachytene exit (Fig 6F and 6H; *p* < 0.001 in comparison to wild types), despite their abundant presence earlier (Fig 6B–6E). Collectively, these data support dual roles for ZHP-4 in RMH-1 dynamics. Our data suggest that wild-type levels of early RMH-1 foci can form in the absence of ZHP-4, however, ZHP-4 is then required to stabilize or localize RMH-1 at early JMs that will become either COs or NCOs, and later at designated crossover sites where the final intermediate will be resolved as a crossover.

### *zhp-4* is required for acquisition of markers of crossover designation

In addition to RMH-1, the crossover-designated sites at mid-late pachytene stages also colocalize with ZHP-3, MSH-5, and COSA-1 [11,12]. *zhp-4(vv96)* mutants displayed punctate staining of ZHP-3 on chromosome tracks at late pachytene (Fig 4A), and a *zhp-4(ha*::*vv96)*-tagged variant that phenocopies the genetic mutant (Materials and Methods) adopted a pattern similar to the ZHP-3 localization in the *zhp-4*(*vv96)* background (S5 Fig). Given that *zhp-4(vv96*) mutants exhibit evidence of significant levels of bivalent formation and crossing over (Fig 3), these data suggest that the ZHP-4^*vv96*^ mutant protein can associate with crossover pathway intermediates in the absence of its contiguous recruitment to the SC to facilitate crossing over.

**Fig 7:**
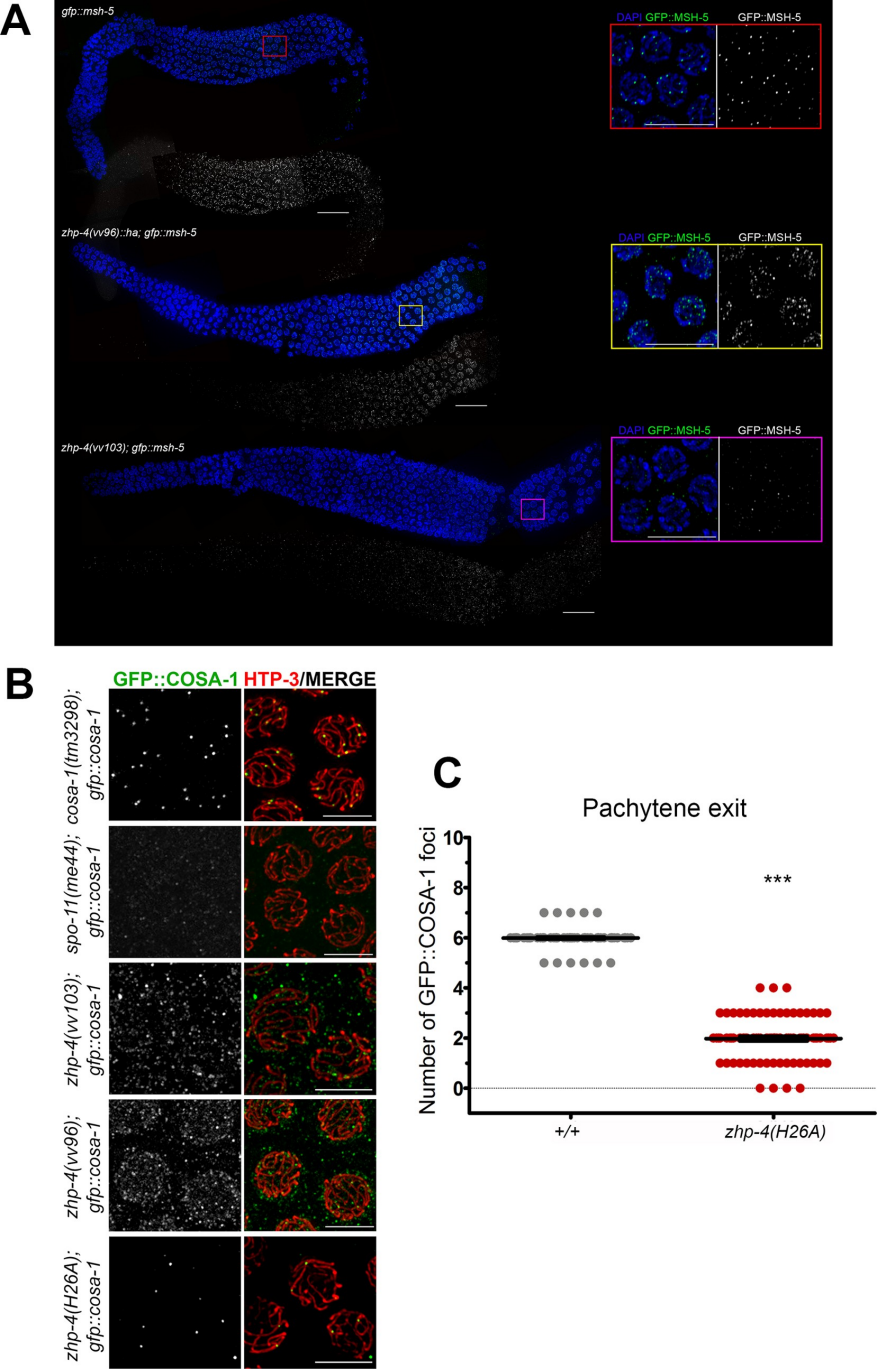
*zhp-4* mutants are defective in acquiring late crossover markers. (A) Whole gonad immunostaining with DAPI (blue) and α-GFP (green) antibody marking GFP::MSH-5 in the indicated genotypes. Pachytene progression proceeds from left to right and insets show magnifications of late pachytene stage nuclei from the corresponding genotype. In wild types, abundant small MSH-5 foci appearing in early/mid-pachytene are reduced to ~ 6 per nucleus by late pachytene, while no foci can be detected above background at any stage in *zhp-4(vv103)* mutants. In *zhp-4(vv96)* mutant germlines MSH-5 foci appear with delayed kinetics and aberrant morphology (of varying sizes and intensities that preclude accurate quantitation); at late pachytene, ~10–25 foci of varying sizes and intensities remain, and occasionally appear in short stretches. Scale bars 10 μm. (B) Immunolocalization of GFP::COSA-1 (green) and HTP-3 (red) in late pachytene nuclei germlines of the indicated genotypes. *zhp-4(vv103)* and *zhp-4(vv96)* mutants fail to form the ~6 bright COSA-1 foci observed in wild types and instead form foci of varying intensities and sizes that do not colocalize with axes, appear as background, or weakly nuclear. *zhp-4(H26A)* mutants form 1–3 foci that appear on synapsed chromosome axes. Scale bars 5 μm. (C) Scatter plots showing the quantification of GFP::COSA-1 foci in pachytene exit stage nuclei (the last row of nuclei before diplotene) of the indicated genotypes. Medians are shown as thick black horizontal bars +/- interquartile range (Mann-Whitney test, *** *p*<0.001).

We next examined the localization of MSH-5, a component of the pro-crossover MutSγ complex, that is required for formation of the obligate crossovers from a population of earlier recombination intermediates. Abundant early MSH-5 foci form during mid-pachytene where they colocalize with a subset of RMH-1 foci and finally mark the six crossover sites in late pachytene stages [11,12]. In the absence of ZHP-4, only a few sporadic small MSH-5 foci that did not colocalize with chromosome tracks could be detected (Fig 7A). Given the correlated deficit of RMH-1-marked recombination intermediates in *zhp-4(vv103)* mutant germlines, these data suggest that MSH-5 recruitment to a potential crossover intermediate may require an RMH-1 processed JM and/or that the two proteins also co-operate in one anothers’ localization/stabilization at these sites. In *zhp-4(vv96)* hypomorphs, abundant MSH-5 foci formed in mid-late pachytene, however, these foci were of varying sizes and intensities that precluded accurate scoring and ultimately did not reduce to the ~6 bright foci observed in wild types. Given that RMH-1-marked recombination intermediates are fewer or more unstable in *vv96* mutants, a likely consequence is that their loss translates to impaired MSH-5 recruitment/stabilization [12], in turn leading to the reduced crossing over and chiasma defects observed in these mutants. Furthermore, no bright wild-type-like COSA-1 foci were detected on chromosome tracks in the germlines of *vv96* and *vv103* mutants (Fig 7B). Its localization was instead reduced to a faint and diffuse signal punctuated by numerous foci of variable sizes and intensities that occasionally overlapped with the synapsed chromosomes, but were often extranuclear. This localization differed from the few faint foci observed in *spo-11* mutants that do not initiate meiotic recombination and may reflect protein interactions outside of the context of crossover formation. Based on these results we conclude that COSA-1-mediated steps in crossover designation require ZHP-4 function.

**Fig 8:**
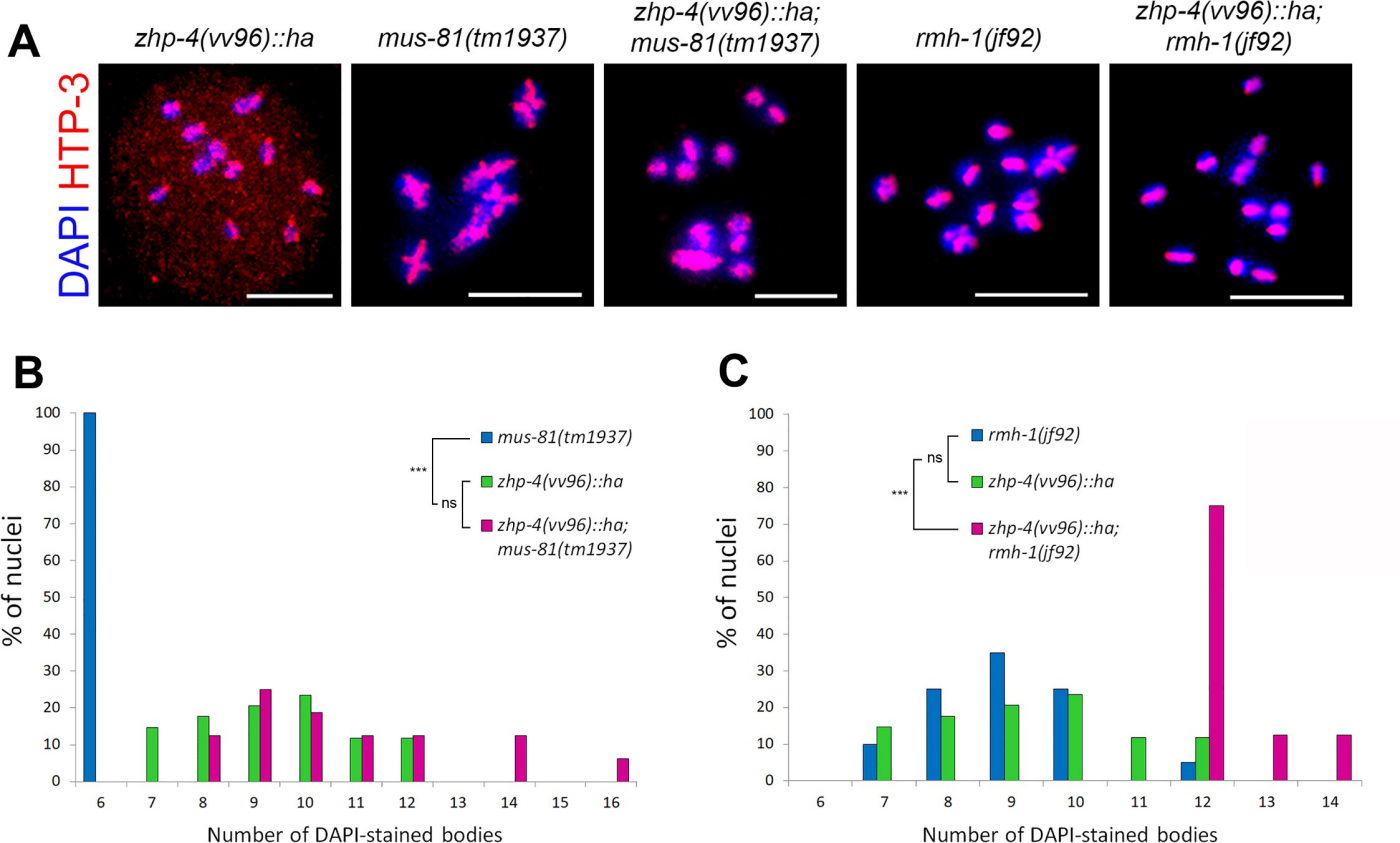
Rare chiasmata observed in *zhp-4(vv96)* mutants are *rmh-1* but not *mus-81* dependent. (A) Representative oocytes from the indicated genotypes showing diakinesis nuclei stained with DAPI (blue) and HTP-3 (red). (B) Quantification of the DAPI bodies in diakinesis nuclei (-1 and -2 oocytes) from germlines of the indicated genotypes. *mus-81* mutants invariably exhibit 6 DAPI structures, whereas the distributions in *zhp-4;mus-81* double mutants is not different from *zhp-4* single mutants mutation (*p*>0.05). (C) Quantification of the DAPI bodies in diakinesis nuclei (-1 and -2 oocytes) from germlines of the indicated genotypes. *rmh-1* mutants show a distribution not different from *zhp-4* mutants while the double mutants have an average of about 12 univalents, significantly different from either single mutant distribution (statistical test Kruskal-Wallis followed by Dunn’s multiple comparison test, ns = not significant *p*>0.05, *** *p*<0.001. Nuclei scored: *zhp-4(vv96)* n = 34, *mus-81(tm1937)* n = 22, *zhp-4(vv96);mus-81(tm1937)* n = 16, *rmh-1(jf92)* n = 20, *zhp-4(vv96);rmh-1(jf92)* n = 16. Scale bars 5 μm.

The pro-crossover factors that showed disrupted localization to late crossover intermediates in *vv96* mutants participate in the resolution of recombination intermediates into class I crossovers that show interference (the reduced probability that a second crossover forms in the vicinity of the first). An alternative class II pathway that includes the MUS-81 structure-specific endonuclease is required for a small subset of meiotic crossovers that are noninterfering [44] and we investigated whether the bivalents that formed in *vv96* mutants depended on the activity of MUS-81 or RMH-1 (Fig 8). The diakinesis nuclei of *zhp-4(vv96); mus-81(tm1937)* double mutants did not show significantly different distributions of DAPI figures in comparison to *vv96* single mutants (*p* > 0.05), indicating that the residual chiasmata observed in *zhp-4(vv96)* mutants do not required *mus-81*. In contrast, an average of 12 DAPI figures and no bivalents were observed at diakinesis in *zhp-4(vv96); rmh-1(jf92)* double mutants (Fig 8B and 8C), indicating that bivalent formation in *vv96* mutants is dependent on RMH-1. Furthermore, the residual chiasmata observed in *rmh-1* single mutants was in turn dependent on *zhp-4*, suggesting possible co-dependent functions in crossover formation. In summary, our collective results suggest that ZHP-4 is first required for the stabilization of RMH-1 at early recombination intermediates; this event either generates a crossover intermediate recognized by pro-crossover factors or stabilizes those factors at the site (or both), leading to resolution of the intermediate into a crossover at pachytene exit and its transition into a chiasma.

### ZHP-4 is required for crossover-triggered remodelling of bivalents

Characterization of the severe hypomorph *zhp-4(vv96)* revealed a functional paradox: although the levels of embryonic lethality and frequency of males in the self progeny did not differ from the null allele (*p* > 0.05, Fig 1B and 1C), *zhp-4(vv96)* mutants exhibited surprisingly substantial levels of both bivalent structures and of crossovers as measured by genetic exchange (Fig 3A, 3B and 3D) despite the defects in acquiring late-pachytene stage RMH-1, MSH-5 and COSA-1 foci that mark sites of the obligate crossovers in wild-type.

**Fig 9:**
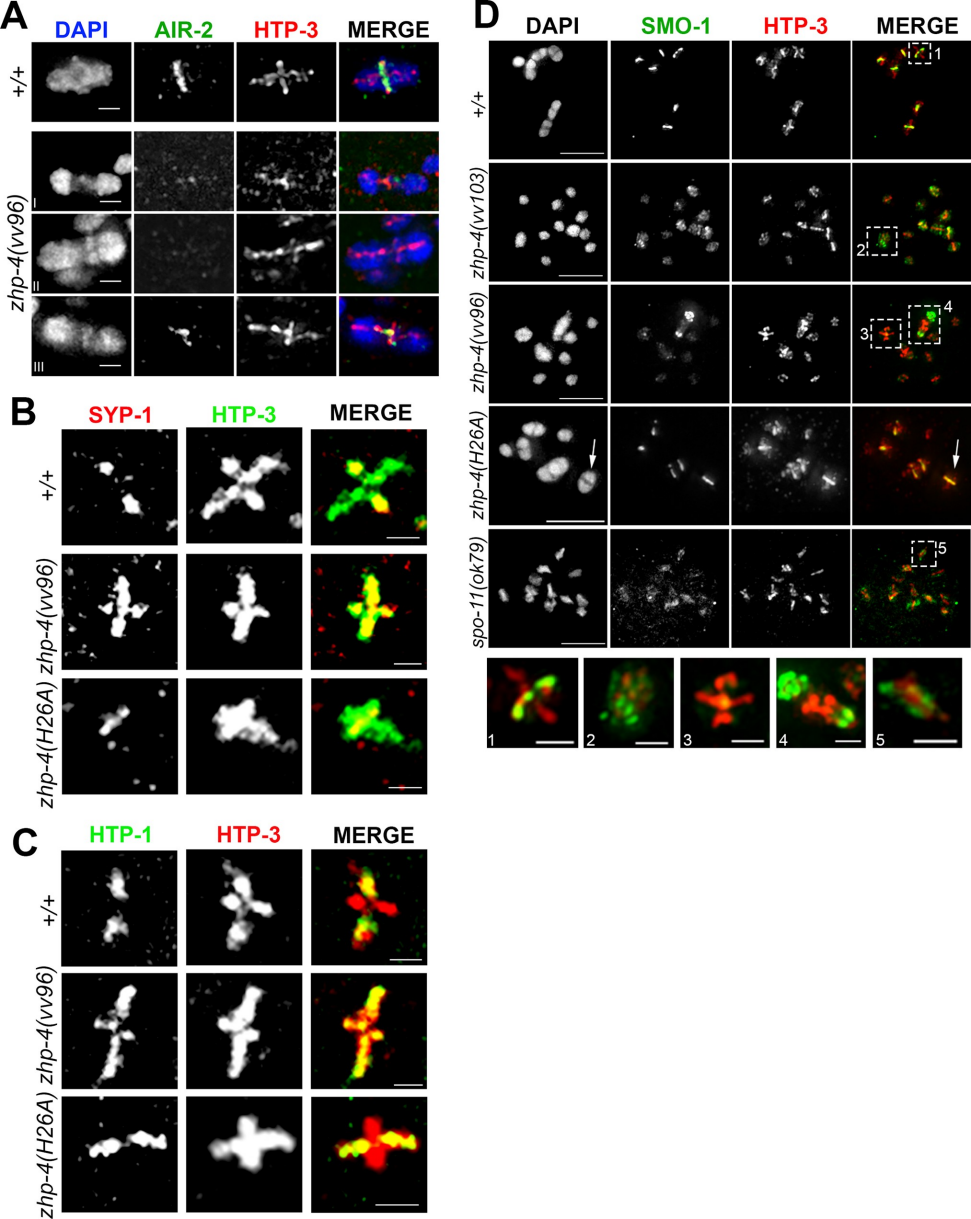
Diakinesis bivalents fail to undergo remodelling in *zhp-4* mutants. (A) Representative images of wild-type and *zhp-4(vv96)* diakinesis nuclei stained with α-AIR-2 (green) and α-HTP-3 (red). Wild-type bivalents exhibit the axial element HTP-3 in a cruciform shape (defining both the long and short axial arms) while AIR-2 is restricted to the short arm. AIR-2 localization in *zhp-4(vv96)* mutants is variable and it can be detected on one HTP-3 axis of a well-formed bivalent structure (bivalent), or be undetectable on an aberrant structure (tethered). Scale bars 1 μm. (B) SC component SYP-1 (red) and HTP-3 (green) immunolocalization in diakinesis nuclei showing representative bivalent structures. SYP-1 localization is restricted to the short arms of the bivalent in wild-type and *zhp-4(H26A)* mutants, but is detected along both long and short arms in the *zhp-4(vv96)* mutants. Scale bars 1 μm. (C) α-HTP-1 (green) and α-HTP-3 (red) staining of diakinesis nuclei showing axis component HTP-1 restriction to the long arm of the wild-type and *zhp-4* ring mutant bivalent while it is continued association with the short and long arm of the bivalents in *zhp-4(vv96)* mutants. Scale bars 1 μm. (D) -1 diakinesis oocyte (the last oocyte of the germline prior to entry into the spermatheca) stained with α-SMO-1 (green; the *C*. *elegans* SUMO ortholog) and α-HTP-3 (red), with magnified insets of individual chromosomal figures from individuals of the indicated genotypes (1–5). SMO-1 localizes to the short arms of bivalents in wild types, a pattern dependent on CO formation since SMO-1 remained associated with chromatin in *spo-11(ok79)* mutants. In both *zhp-4(vv103)* and *zhp-4(vv96)* nuclei, SMO-1 remained associated with the chromatin, even on bivalents assessed by the volume of the chromosome structures and HTP-3 staining. In *zhp-4(H26A)* mutants, SMO-1 localizes to the short arms axis of all bivalents and remains with the chromatin of the univalents. Magnifications of in lower row show (1) wild-type bivalent with SMO-1 localized to the short arms, (2) a bivalent in *zhp-4(vv103)* mutants with SMO-1 localized to the chromatin, (3) well-formed bivalent (as assessed by HTP-3 staining) in *zhp-4(vv96)* mutants without SMO-1 on the short arms, (4) example of tethered bivalent with enriched localization of HTP-3 at the tether and persistent chromatin localization of SMO-1, (5) a univalent in *spo-11(ok79)* mutants exhibiting SMO-1 localization with chromatin. Scale bars for main panel 5 μm; bivalent magnifications (1–5) 1 μm.

Close examination of chromosomes in the diakinesis nuclei of *zhp-4(vv96)* mutants revealed an unexpected phenotype; the occasional appearance of well-condensed bivalent-like structures that had separated chromosomes and were tethered to one another by a chromatin mass, with the axial element HTP-3 often congregated within (Fig 9A). While abnormal bivalent morphology in which chromosomes linked by chromatin bridges has been observed in mutants that disrupt Holliday junction resolution and crossover intermediate processing, the chromatin linkages in these cases appear more thread-like and do not contain axis components [12,45]. The anomalous diakinesis structures observed in *zhp-4(vv96)* mutants appeared in a SPO-11-dependent manner, indicating that they were the outcome of a meiotically-programmed DSB intermediate (Fig 3A and 3B), and are for simplicity referred to as “tethered bivalents”. To characterize this disruption, we probed the bivalents observed in *vv96* mutants for evidence of the remodelling associated with chiasma formation (Fig 9), including restriction of the AIR-2 kinase (aurora B kinase; [46,47]) and SC component SYP-1 to the short arms of the bivalent [32], and the meiotic sister chromatid cohesin regulator HTP-1 to the long arms [33]. In *vv96* mutant oocytes at diakinesis, bivalents (as assessed by size and cruciform staining of the axis marker HTP-3) failed to appropriately remodel (Fig 9A–9C) and instead exhibited SYP-1 (13/13 bivalents) and HTP-1 (11/12 bivalents) along the axes of both the long and short arm. *zhp-4(vv96)* mutants similarly displayed disruptions to AIR-2 localization that interfered with quantitation; these included failure to localize or its appearance in chromatin masses between the tethered bivalents. Taken together, these results indicate that the crossovers forming in *vv96* mutants are defective in triggering the associated remodelling of factors implicated in chromosome segregation, or that this triggering is not executed in *vv96* mutants.

To further investigate the functional implications of this localization defect, we next examined the localization of the single *C*. *elegans* SUMO ortholog whose conjugation to target proteins promotes chromosome alignment at metaphase I [48]. SMO-1 localizes to the chromatin of germline nuclei and then to the axes of the bivalent short arms in late diakinesis stage oocytes ([48]; S6 Fig); this dynamic localization is recombination dependent, since SMO-1 remains diffusely associated with chromatin rather than restricted to HTP-3-marked chromosome axes in *spo-11(ok79)* mutants (Fig 9D). In both *zhp-4(vv103)* null *and zhp-4(vv96*) hypomorphic mutants, SMO-1 localizes appropriately to chromatin of mitotic and prophase chromosomes and remains associated with the chromatin of univalents present in late-stage diakinesis nuclei, similar to the localization observed in *spo-11* mutants (Figs 9D and S6). In contrast to wild types, however, SMO-1 remained associated with the chromatin of diakinesis bivalents in *vv96* mutant nuclei and with the chromatin of a rare bivalent observed in a *vv103* mutant nuclei (Fig 9D.2-3). In the case of *vv96* mutants, SMO-1 also occasionally appeared enriched in the chromatin at the ends of tethered bivalents (Fig 9D.4). Consequently, we conclude that the crossovers that form in *vv96* or *vv103* mutants can give rise to bivalent-like figures, however, these exhibit structural anomalies and are defective in the chromosome remodelling that normally accompanies crossover formation and chiasma emergence. In contrast, the bivalents that form in the diakinesis nuclei of *zhp-4(H26A)* mutants show appropriate localization of SYP-1, HTP-1, and SMO-1 and no tethered bivalent phenotypes (Fig 9B–9D; no tethered bivalents observed in 86 diakinesis nuclei), suggesting that the reduced number of crossovers that form are competent to trigger chromosome remodelling, correlated with improved embryonic survival (Fig 1B).

To investigate the possible functional consequences of the formation of aberrant bivalent structures in *zhp-4(vv96)* mutants, we examined chromosome congression and segregation at the metaphase plate in meiosis I. In wild-type, bivalents align at the metaphase plate I between overlapping microtubule bundles that form channels through which the chromosomes move during segregation [49,50]. At metaphase I, *zhp-4(vv96)* oocytes displayed a spectrum of phenotypes from occasional wild-type spindle organization to stray chromosomes and abnormal spindle morphology as assessed by disorganized microtubule channels (S7 Fig). These structural defects in spindle assembly correlated with aberrant segregation behaviour at anaphase I; while in wild-type oocytes two distinct masses of chromatin were separated by the microtubule channels, the chromosomes of some *zhp-4(vv96)* mutant oocytes appeared unresolved and tangled while being pulled to the poles (S7 Fig). Given the presence of bivalents in *zhp-4(vv96)* mutants, we favour the interpretation that the crossovers that form in the absence of full ZHP-4 function do not trigger the bivalent remodelling associated with preparation for segregation and consequently lead to the same level of embryonic lethality and X chromosome nondisjunction as observed in *zhp-4(vv103)* null mutants. These results are consistent with a model in which crossover and chiasmata formation are genetically separable events that require ZHP-4 for the physical transformation of crossovers into the chiasmata capable of directing chromosome segregation at the meiotic spindle.

### ZHP-4-mediated crossover maturation correlates with segregation competency

*zhp-4(H26A)* mutants shared several similar phenotypic features with *zhp-4(vv96)* mutants, including faint and punctate SC localization and significant levels of genetic crossing over. In contrast to *zhp-4(H26A)* mutants, however, *zhp-4(vv96)* mutants 1) did not form ZHP-3/4 late pachytene foci, despite the presence of genetic crossovers, 2) contained aberrant tethered bivalents in diakinesis nuclei which were not observed in *zhp-4(H26A)* mutants, and 3) exhibited significantly higher levels of embryonic lethality (Fig 1B). Given that the *zhp-4(vv96)* mutant defects correlated with disrupted RMH-1 stabilization at recombination intermediates and failure to retain/recruit markers of designated crossovers/chiasmata, we investigated these processes in ZHP-4 RING domain mutants. *zhp-4(H26A)* mutants exhibited early RMH-1 foci dynamics that resembled those observed in *zhp-4(vv96)* mutants (Fig 6A–6E, *p* > 0.05 for all except *p* < 0.05 for C), including: 1) their appropriate appearance at very early pachytene, 2) a delay in their accumulation, and 3) the appearance of wild-type levels of RMH-1-marked recombination intermediates at mid-late pachytene stages (Fig 6D, *p* > 0.05 vs. WT). However, in nuclei entering the pachytene exit stage in which designated crossover/chiasma markers emerge, the majority of *zhp-4(H26A)* nuclei exhibited 1–3 RMH-1 foci/nucleus (consistent with the appearance of 1–3 ZHP-4 foci in the RING mutant), while none were detected in *zhp-4(vv96)* mutants (Fig 6F–6H, *p <* 0.05 compared to *vv96* mutants). The presence of these RMH-1 foci at this late stage suggests that the RING domain mutant is competent to form a reduced number of the crossover intermediates that are observed in wild types. To investigate this possibility, we next examined *zhp-4(H26A)* pachytene exit stage nuclei for the appearance of COSA-1 marked-crossover intermediates, which could not be detected on chromosomes above background levels in *zhp-4(vv96*) mutants (Fig 7B). *zhp-4(H26A)* mutants exhibited levels of COSA-1 foci formation similar to that observed for RMH-1 focus formation at late pachytene (median of 2; Fig 7C, *p <* 0.001 in comparison to wild types), and in both cases the bright COSA-1/RMH-1 foci appeared on HTP-3-marked synapsed axes as observed in wild types. Furthermore, well-formed bivalents with respect to DNA condensation and the localization of SYP-1/SMO-1 to the short arm and HTP-1 to the long arm appeared in *zhp-4(H26A)* mutants at late diakinesis, indicating that the genetic crossovers detected (Fig 3D) correlate with the presence of designated crossover markers and appropriate remodelling of the bivalent (Fig 9B–9D, white arrow). The association of late pro-crossover factors with the designated crossover site correlated not only with bivalent remodelling, but also with the ability of the chiasmata to direct segregation as evidenced by the lower embryonic lethality observed in *zhp-4(H26A)* mutants in comparison to *zhp-4(vv96)* mutants (Fig 1B). These results strongly suggest that the crossover intermediates that do acquire RMH-1, ZHP-4 and COSA-1 at very late pachytene stages in *zhp-4(H26A)* mutants define crossovers competent to form chiasmata and trigger the bivalent remodelling that is required to ensure accurate chromosome segregation. Consistent with this interpretation, *zhp-4(vv96)* mutants are competent for the formation of RMH-1/MSH-5 foci in mid-pachytene stages and reduced levels of crossing over; however, the crossovers that do form are not cytologically visible as foci containing the chiasmata markers, and correlate with defects in chromosome remodelling and spindle function. We propose that ZHP-4 acts in concert with ZHP-3 to stabilize RMH-1 at early recombination intermediates to foster the formation of an early crossover intermediate competent for negatively regulating meiotic DSB induction and association with other pro-crossover factors like MSH-5 and COSA-1. Our analysis suggests that the ZHP-4-mediated stabilization/recruitment of the pro-crossover complex at designated crossover sites by late pachytene is required to convert the DNA exchange events into the chiasmata solely capable of triggering bivalent remodelling in preparation for meiotic spindle assembly and chromosome segregation.

## Discussion

### ZHP-3/4 are RNF212 orthologs that are targeted to the SC through the ZHP-4 RING domain

A further evolution in our understanding of E3 ligases has been the observation that some can function as heterodimers, including the Ub ligases BRCA1-BARD1 [51–53] and SUMO-directed Ub ligases Slx5-Slx8, [54–57]; however, no such examples have yet emerged for SUMO E3 ligases. Here, we have shown that ZHP-3/4 colocalize throughout meiotic prophase and that their localization to the SC and to designated CO sites is interdependent, suggesting a cooperative activity. Although the ZHP-3 RING finger is competent to support the formation of rare COs, the RING finger domain of ZHP-4 is the preferred contestant in localizing the complex to the SC since ZHP-3 RING activity can only be detected in its absence. This difference in terms of requirement between the two RING finger domains mirrors other examples of heterodimeric Ub E3 ligase complexes: in the case of BRCA1 and BARD1, BRCA1 is the ‘active’ partner while BARD1, the ‘inactive’ partner, stabilizes the complex *in vivo* (reviewed by [40]). Although ZHP-3/4 has been suggested to act as a heterodimeric E3 SUMO ligase [29], such a function is not supported by the phenotype of mutants in the single nematode SUMO gene, which form bivalents rather than the predicted univalents [22,58]. An outstanding question is whether ZHP-3/4 is a ubiquitin ligase, and if so, if it could perform a structural role at crossover sites that becomes a catalytic role in restructuring the resulting bivalent.

The family of RNF212-like orthologs have differences and similarities in terms of localization and their relationship with DSB formation and SC assembly that largely reflects the relationship between recombination initiation and SC initiation in each organism. Recent studies have revealed that *S*. *cerevisiae* Zip3 and mouse RNF212 recruitment to meiotic chromosomes occurs in two distinct modes, one being DSB-dependent foci, and the other requiring SC formation for localization [18,25,59], indicating a DSB-independent mechanism of a CO-promoting factor. In the divergent world of plants and filamentous fungi, the single Zip3-like protein (HEI10) similarly localizes in pachytene to the SC central regions and is then restricted to detectable foci at late stages that correspond to sites of both COs and chiasmata [23,24,26].

Although SC initiation is independent of DSB formation in *C*. *elegans*, ZHP-3/4 exhibit two patterns of localization that grossly reflect the localization of RNF212 in mice: 1) ZHP-3/4 initially localize along the SC in an SC-dependent (and SPO-11-independent) manner, and 2) are restricted to a few sites of crossovers (~ 6 foci) that at late pachytene stages are dependent on both the SC and SPO-11 (S2C Fig; [21]). While in *Drosophila*, the RNF212-like ortholog Vilya is required for DSB formation to occur [28], our study indicates that ZHP-3/4 are not required for the initiation of meiotic DSB formation (DSBs form in the absence of the proteins) and SC assembly (Figs 5 and S1C; [21]). Instead, ZHP-3/4 are required to foster the transition of a limited number of crossover intermediates into *bona fide* crossover entities, a function which includes negatively regulating meiotic DSB induction once crossover intermediates have been formed. Overall, a common and recurring feature of all RNF212-like orthologs is their localization to the SC either as continuous linear stretches, or as a population of small foci that later emerge as larger discrete foci that mark crossing over sites. Given these localization dynamics across species with differential requirements for synapsis initiation (DSB dependent or independent), our results are consistent with an intimate relationship between RNF212 family members and the SC from the earliest stages of recombination initiation that supports relatively rare events to go forward as the crossovers that will support chromosome segregation.

### ZHP-3/4 are required for the stabilization of RMH-1 at early JM intermediates

In this study, we have shown that *zhp-3/4* are required at distinct stages in the transition of JMs to chiasmata; at mid-pachytene stages where they promote the formation of an RMH-1-mediated JM competent to recruit pro-CO factors and at pachytene exit stages where they are required for the transition from crossover-designated sites to chiasmata. Since DSB formation, as visualized by RAD-51, loading initiates on time and at robust levels in *zhp-4* mutants (see below), the deficit of chiasmata observed in the diakinesis oocytes indicates a defect in post-initiation/strand exchange process. At mid-pachytene stages RMH-1 cooperates with BLM (nematode HIM-6) to promote crossover outcomes at JMs and repair the remaining recombination intermediates as noncrossovers [12,34]. We observed that the first population of RMH-1 appears on time and at appropriate levels in *zhp-4* mutants, indicating that recruitment of RMH-1 to early recombination intermediates does not require ZHP-4; at early/mid-pachytene, however, both null and hypomorphic mutants exhibited severe defects in presenting wild-type numbers of RMH-1 foci, collectively consistent with the interpretation that ZHP-4 is required for stabilization of RMH-1 at JM rather than recruitment *per se*. The failure to stabilize RMH-1 at the early-mid-pachytene stage in *zhp-4* mutants correlates with altered dynamics of other markers of recombination progression. First, RAD-51-marked recombination intermediates showed dramatically increased levels from the transition zone/earliest pachytene stages that followed wild-type-like kinetics of appearance and disappearance, suggesting an overall increase in meiotic recombination initiation rather than a defect in RAD-51 turnover. Second, the mid-pachytene MSH-5 foci that colocalize with RMH-1 at JMs in wild-type fail to form in the absence of *zhp-4* and in *vv96* hypomorphs appear with delayed timing and altered morphologies. Both phenomena are most parsimoniously explained as outcomes of a single event; *zhp-3/4* is required for stabilization of RMH-1 at early/mid-pachytene stages to produce a JM intermediate that can signal the end to meiotic DSB initiation, leading to stabilization of interhomolog intermediates, CO designation, and crossover formation. Consequently, we favour the interpretation that the loss or reduction of crossing over observed in *zhp-4* mutants originates in the failure to form a stable RMH-1-associated JM intermediate that can progress into the crossover pathway, and is instead repaired as an NCO.

### ZHP-3/4-mediated accumulation of pro-crossover factors at designated CO sites is required for formation of chiasmata competent to direct segregation

In addition to the early role of *zhp-3/4* in the crossover pathway, our analysis of the *zhp-4* mutants revealed a genetically separable role for ZHP-4 at pachytene exit stages in the designation of crossover intermediates destined to become chiasmata. *zhp-4(vv96)* and *zhp-4(H26A)* RING mutants share a similar early/mid-pachytene phenotypic profile: in both cases, ZHP-3/4 do not show localization along the SC, RMH-1 appears with delayed kinetics that ultimately reaches wild-type levels, detectable genetic crossing over occurs, and similar distributions of bivalent structures appear at diakinesis. At late pachytene stages when pro-crossover markers are restricted to CO sites, *zhp-4(H26A)* RING mutants exhibit 1–3 bright foci appropriately marked by RMH-1, ZHP-4, and COSA-1, suggesting that the earlier problems in RMH-1 dynamics are overcome to generate a reduced number of wild-type crossovers. In *zhp-3(H25A); zhp-4(H26A)* double RING mutants, these ZHP-4-marked foci fail to form, a phenotype which is accompanied by loss of bivalent formation (Figs 3C and 4B). This suggests that crossing over in ZHP-4 RING mutants is dependent on ZHP-3 and likely reflects a scenario in which the ZHP-3 RING domain is sufficient to support highly reduced ZHP-3/4 localization to chromosomes where its function is unaltered. In the case of *vv96* mutants, however, crossing over and bivalent formation are not accompanied by the appearance of crossover-designated sites as defined by RMH-1/COSA-1 focus formation, indicating that CO designation and formation are separable events. The failure to form late RMH-1/COSA-1 foci in *vv96* mutants correlates with the appearance of anomalous bivalent structures unique to *zhp-4(vv96)* mutants and well-formed bivalents that fail to exhibit the CO-directed remodelling associated with preparation for segregation. These chromosomes often show gross defects in alignment and congression at the metaphase I spindle and remain entangled at anaphase I, consistent with the chromosome segregation defects and high embryonic lethality observed in *zhp-4(vv96)* mutants. Similarly, *zhp-3*::*gfp* mutants (the *gfp* construct does not fully rescue the null mutant phenotype at standard culture temperatures [22]) display a competency for crossover formation that is nevertheless accompanied by unexpectedly high levels of embryonic lethality and X-chromosome nondisjunction, suggesting that the significant levels of crossing over in the presence of altered ZHP-3 function does not always guarantee chiasmata formation and bivalent remodelling [22]. In many organisms, the correct placement of crossovers on the chromosomes has been proven to be pivotal for promoting segregation; COs at the centromeres or ends of chromosomes are less effective at ensuring disjunction (reviewed by [3]). In the case of *zhp-4* mutants, an argument can be made that the inability of the COs to ensure accurate segregation is a consequence of their displacement to disjunction-ineffective regions. However, the nematode *rec-1* mutant redistributes a wild-type number of COs in a pattern reflecting the physical map without compromising chromosome segregation [60], indicating that CO redistribution *per se* is not sufficient to provoke nondisjunction.

The early prophase localization of the ZHP-3/4 complex presents an elegant solution to the requirement for *zhp-3/4;* they function in promoting CO intermediate formation, while being dispensable for initial pairing or meiotic DSB induction [39,61], The SC is required for crossing over and recruitment of the complex to the structure and concentrates its activities in proximity to nascent HR intermediates from the earliest time point that JMs can enter the crossover pathway. An outstanding question that remains is the function of ZHP-3/4 at the crossover-designated sites that appear at late pachytene stages. The behaviour of *vv96* mutants suggests that lost or disrupted retention of late pro-crossover markers at designated sites does not necessarily abolish crossing over, but does disrupt some aspect of CO formation that has functional consequences for the resulting bivalent during chromosome segregation. Recent work on the architecture of nematode recombination complexes and their relationship to the SC during meiotic prophase has observed that CO/NCO outcomes are visibly manifested at late pachytene stages [34]. HR repair proteins are lost from NCO sites (presumably indicative of completed repair) and pro-crossover MSH-5, COSA-1, and BLM (RMH-1-interacting nematode protein HIM-6; [12]) appear at CO-designated sites in the context of central region components of the SC that envelop them in a bubble-like structure. Although the function of this structure is not known, an intriguing possibility is that it reflects an enzymatic caging which can concentrate the pro-CO activities within and protect the CO intermediate from the NCO activities taking place outside. Since ZHP-3/4 are dependent on central region SC components for their localization, it is possible that their function in this compartment is to stabilize the pro-crossover factors until desynapsis at diplotene frees the double Holliday junction (dHJ) precursor for resolution into a crossover. In this context, the consequences of the inability of *vv96* mutants to form these late pro-crossover factor-enriched sites may result in premature exposure of the dHJ to resolvases that temporally uncouple crossover formation from chiasma emergence and regulated swapping or remodelling of the axes to which the involved DNA is tethered [62]. Such a function may explain the fact that *zhp-4(vv96)* mutants are in part marked by the appearance of diakinesis bivalents that are tethered by chromatin masses engaged with axis components, in addition to other bivalent structural anomalies that are suggestive of perturbed coordination between dHJ resolution and CO-triggered chromosome morphogenesis. We speculate that the failure to coordinate these events may distinguish a genetic crossover at the DNA level from a chiasma competent to direct chromosome segregation by disrupting axis exchange and/or patterning of sister chromatid cohesion.

## Materials and methods

### Genetics

*C*. *elegans* strains were cultured under the conditions described by Brenner [63] and all experiments were conducted at 20°C. The N2 var. Bristol strain was used as a wild-type reference and the following mutations and rearrangements were used: *zhp-3 (jf61*::*unc-119+) /hT2 I*. *meIs8 [Ppie-1*::*gfp*::*cosa-1 + unc-119(+)] II; cosa-1(tm3298) III*. *jfsi38 [gfp*::*rmh-1 cb-unc-119+] II*. *dpy-18(e364) unc-25(e156) III*. *spo-11(ok79)/nT1 IV*. *spo-11(me44)/nT1 IV*. *dpy-3(e27) unc-3(e151) X*. *rmh-1(jf92[M01E11*.*3*::*unc-119+]) I*. *mus-81(tm1937) I*. *syp-2(ok307) V*. *msh-5*::*gfp IV*.

### Measurement of embryonic lethality and incidence of male progeny

Hermaphrodites were singled at L4 stage and transferred daily to fresh plates for three consecutive days. The number of eggs of each hermaphrodites was recorded immediately after each transfer; in the last plate, the number of eggs was recorded 24 hours after transfer. The number of hermaphroditic and male progeny were scored three days later. Embryonic lethality rate was calculated as the total number of surviving progeny divided by the total number of eggs. Incidence of males was calculated as the number of males divided by the total number of surviving progeny.

### Genetic recombination

Recombination was assayed using visible markers by crossing *zhp-4(vv96)/+* males with *zhp-4(vv96) V; dpy-3(e27) unc-3(e151) X* and *zhp-4(vv96) V; dpy-18(e364) unc-25 (e156) III* hermaphrodites. Similarly, *zhp-4(H26A)* males were crossed with *zhp-4(H26A) V; dpy-3(e27) unc-3(e151)* hermaphrodites. NonUnc, nonDpy F1 cross progeny were picked and allowed for self-fertilize. F1s that were homozygous for *zhp-4(vv96)* and *zhp-4(H26A)* respectively were identified by embryonic lethality (Emb) and high incidences of males (Him) in the F2 progeny. For wild-type, 1973 (1334 wild-type and 639 recombinants) F2 progeny were scored from 10 *dpy-3 unc-3/+ +* and 3451 (2966 wild-type and 485 recombinants) F2 progeny were scored from 15 *dpy-18 unc-25/+ +* heterozygotes. For *zhp-4(vv96)* mutants, 507 (437 wild-type and 70 recombinants) F2 progeny were scored from 37 *dpy-3 unc-3/+ +; zhp-4(vv96)/zhp-4(vv96)* and 815 (710 wild-type and 105 recombinants) F2 progeny were scored from 67 *dpy-18 unc-25/+ +; zhp-4(vv96)/zhp-4(vv96)*. For *zhp-4(H26A)* mutants, 1165 (913 wild-type and 252 recombinants) F2 progeny were scored from 57 *dpy-3 unc-3/+ +; zhp-4(H26A)/zhp-4(H26A)* heterozygotes and 558 (524 wild-type and 34 recombinants) F2 progeny were scored from 35 *dpy-18 unc-25/+ +; zhp-4(H26A)/zhp-4(H26A)* heterozygotes. Recombination frequencies were calculated as previously described [61], where the frequency (*p*) between two markers was calculated using the formula *p* = 1 - (1 - 2R)^1/2,^ where R is the number of visible recombinant individuals divided by the number of total progeny. The number of total progeny for the hermaphrodite was calculated as 4/3 X (number of Wts + one recombinant class) to compensate for the inviability of the double homozygote class. Both classes of recombinants were used in the calculations.

### Identification and generation of *zhp-4* mutants using CRISPR-Cas9 mutagenesis

The *zhp-4(vv96)* allele was recovered from a “Green Egg” mutagenesis screen (50 mM EMS) that isolated mutants with X-chromosome segregation defects [14]. Cloning of *vv96* revealed a C to T substitution at the 160th codon, which changes the glutamine residue (Q) into a premature stop codon in the coding sequence Y39B6A.16, predicted to be a paralog of ZHP-3 and named ZHP-4 [29]. The wild-type tagged line of *zhp-4(vv117[zhp-4*::*ha])* was generated by Shaolin Li (Gene Editing Services). All the other alleles were generated by directed mutagenesis using CRISPR-Cas9 protocol previously described [64] with the only difference that Cas9 protein was purchased from PNA Bio (CP01-200). For a list of sgRNAs and repair templates refer to S1 Table. In the case of *zhp-4(vv103)* an indel mutation was introduced in the RING-finger domain in front of the last two cysteine residues, which resulted in a frameshift and eventually a premature stop codon. The wild-type sequence is ATTATGTCATCCACCGGAAG-AAG while the mutant sequence is ATTATGTC——CGGAAGAAAG. The mutant *zhp-4(vv96*::*ha)* has been created by the positioning of the tag right before the stop codon introduced by *vv96* mutation. This strain perfectly mimics the *zhp-4(vv96)* untagged worms allowing us to use either of them according to necessity. The ring mutants *zhp-3(vv137[H25A])* and *zhp-4(vv138[H26A])* harbour the following mutations respectively: CAC-to-GCC and CAT-to-GCC, both substituting a highly conserved His to an Ala.

### Generation and purification of ZHP-4 antibody

To raise antibodies against ZHP-4 and avoid cross-reactivity with other RING domain containing proteins, a fragment of 372 base pairs corresponding to the C-terminus of ZHP-4 was cloned into two bacterial expression vectors: pGEX-6p-2, containing the GST tag at the N-terminus (GE Healthcare) and pET28a (Qiagen), to generate an N-terminal 6xHis-fusion protein. Recombinant proteins were purified under native conditions using anti-GST beads (GE Healthcare) and Ni-NTA matrix (Qiagen) respectively following the manufacturer’s instructions. GST::ZHP-4 was used for antibody production in rat and 6xHis::ZHP-4 was used for sera purification (Medimab). ZHP-4 antibody was purified using activated supports according to the manufacturers’ protocols (Affi-Gel 10, BioRad).

### Immunostaining of embryos and germlines

For whole embryo staining, thirty-forty of 24-26h post-L4-staged adults were dissected in 1xPBS, followed by freeze crack using liquid nitrogen and fixation in methanol -20°C for 30 minutes. For whole germline staining, gonads of 24-26-h post-L4 staged adults were dissected in 1XPBS and fixed by 1% paraformaldehyde for 5 minutes, followed by freeze crack and fixation in 100% methanol for 5 minutes at -20°C. After fixation in methanol, slides were washed with PBS-T (0.1% Tween-20) for 5 minutes 3 times. Gonads were then blocked with 1% BSA in PBS-T for an hour and incubated with primary antibodies overnight at 4°C. The following day, slides were washed for 3 times 15-minutes each and then incubated with secondary antibodies for two hours. Afterwards, they were washed 3 times, 15 minutes each. 1μg/μL of DAPI in anti-fading agent (Vectashield) was added onto the slides. Images consisting of 15–20 stacks (of 0.2μm increments), were acquired and processed using a Delta Vision Deconvolution system equipped with an Olympus 1X70 microscope or a Spinning-disc confocal microscope (Leica DMI 6000B inverted microscope equipped with a Quorum WaveFX spinning Disc and EM CCD camera). The following antibodies were used in this study: guinea pig and rabbit α-HTP-3 (1:500–1:750), goat α-SYP-1 (1:1000, gift from M. Colaiacovo), rabbit α-HIM-8 (1:200, Novus Biological, 41980002), mouse α-GFP (1:200, AbCAM ab290), rabbit α-RAD-51 (1:1125), guinea pig α-ZHP-3 (1:750), rabbit α-AIR-2 (1:200), rabbit α-HTP-1 (1:400) rat α-ZHP-4 (1:200), mouse α-HA (1:100, BioLegend, 901513), mouse monoclonal α-SMO-1 6F2 (1:10, DSHB), tubulin-FITC conjugate (1:500, Sigma F-2168), guinea pig α-SUN-1 S8Pi (1:700). For specificity of anti-HA staining see S2B Fig. Secondary antibodies used in this study were: AlexaFLuor 555 goat α-guinea pig (Molecular Probes, A21435) and α-rabbit (Invitrogen, A21429), AlexaFluor 488 goat α-rabbit (Molecular Probes, A11034), AlexaFLuor 488 donkey α-guinea pig, AlexaFluor 555 donkey α-goat (Abcam 150130), AlexaFluor 488 goat α-rat and AlexaFluor 488 goat α-mouse (Jackson ImmunoResearch, 106498), all of which were used in 1:1000 dilution.

### Quantification of RMH-1, COSA-1 and RAD-51

RMH-1 and COSA-1 are both fused with GFP and, following anti-GFP staining, all the foci were scored in each nucleus in the entire pachytene region of each gonad. For representation of the data, the pachytene region was divided into six equal zones and labelled as: early, early-mid, mid, mid-late, late pachytene, and pachytene exit; three gonads were scored for each genotype. For RAD-51 an anti RAD-51 antibody was used for the staining, and foci were scored along the whole gonad of each genotype untill the end of pachytene and then divided into six equal zones (three gonads per genotype were scored).

### rad-54(RNAi)

RNA interference experiments were performed as described previously [65]. In brief, dsRNA was generated using PCR amplification of 946 bp of *rad-54* gene using the primers TTCAGGACGAACGGAGGAAC and TTCCACTGTCCACTGGCATC, followed by *in vitro* transcription with T7 RNA polymerase (Ambion). At 6–8 hours post-L4, very young hermaphrodites were injected and after 2 days processed for cytological analyses. The efficacy of the RNAi was never complete since the injected animals never showed sterility as expected by complete knockdown of *rad-54* [41]. All the eggs laid by injected animals hatched, and their progeny were not sterile; however, cytological analysis of two day post injection animals showed an increase of RAD-51 foci in their germlines until mid-pachytene stage (S4B Fig). Scoring of these foci revealed levels of RAD-51 foci that were comparable to those previously reported [41]. Therefore, our analysis was based on scoring RAD-51 foci in zones 1 through 4 which were affected by *rad-54(RNAi)*.

### Statistics

Distributions of DAPI-stained bodies, RMH-1 foci and COSA-1 foci were statistically tested by Kruskal-Wallis test followed by Dunn’s multiple pairwise coparison tests. The significance of RAD-51 foci scoring was tested by Mann-Whitney U test while embryonic lethality and incidence of males by ANOVA followed by multiple pairwise comparison tests. All calculations were performed with Prism 5 (GraphPad) and *p* < 0.05 was considered significant.

## Supporting information

S1 Table Reagents used for generating mutants by CRISPR-Cas9 mutagenesis. (DOCX)

S1 Fig ZHP-4 function is not required for homolog pairing and SC assembly. (A) Representative gonads from wild-type and *zhp-4* mutant animals show that nuclear morphology as assessed by DAPI staining is not affected in the mutants. Transition zone of each gonad is marked by the white boxes. Scale bars 10μm. (B) Immunostaining for HIM-8 in green (X chromosome pairing center binding protein) show the X chromosomes to be paired in all mid-pachytene nuclei of both *zhp-4* mutants. (C) Representative images of mid-pachytene nuclei immunostained for SYP-1 in green (transverse element of the SC) and HTP-3 in red (axial element of the SC) on wild-type and *zhp-4* germlines showing no defect in SC assembly. Scale bars 5μm.
(TIF)

S2 Fig ZHP-4 is recruited to meiotic chromosomes in an SC-dependent manner. (A) Representative images of different stages of meiotic prophase I in wild-type gonads immunostained for ZHP-4 (green) and HTP-3 (red) antibodies. ZHP-4 is first seen as bright foci at transition zone. Starting from early pachytene, it can be detected along the chromosome tracks. Transitioning from mid to late pachytene, ZHP-4 is restricted to shorter stretches and eventually to six foci per nucleus on average, occasionally shown as short stretches at late pachytene. Some of these foci can be observed at diplotene but they were completely removed by early diakinesis. (B) Representative images of mid-pachytene nuclei of both wild-type and *zhp-4*::*ha* germlines immunostained with anti-HA (green) and HTP-3 (red) antibodies. No specific signal is detected in wild-type germlines, whereas linear tracks of ZHP-4::HA colocalize with HTP-3 in *zhp-4*::*ha* germlines. (C) Immunostaining for ZHP-4 (green) and HTP-3 (red) in wild-type and *syp-2(ok307)* germlines showed that the recruitment of ZHP-4 to meiotic chromosomes is dependent on the SC proteins. Scale bars 5μm.
(TIF)

S3 Fig *C. elegans* ZHP-4 protein has a conserved RING finger structure and belongs to the Zip/RNF212 family. (A) Maximum likelihood tree constructed from a multiple whole protein sequence alignment of *Mus musculus* (Mm) HEI10 and RNF212, *Homo sapiens* (Hs) HEI10 and RNF212, *Oryza sativa* (Os) HEI10, *Arabidopsis thaliana* (At) HEI10, *Caenorhabditis elegans* (Ce) ZHP-3 and ZHP-4, *Drosophila melanogaster* (Dm) Vilya and *Saccharomyces cerevisiae* (Sc) Zip3. Proteins were aligned using MUSCLE and a phylogenetic maximum likelihood tree was constructed using Phylogeny Analysis (http://www.phylogeny.fr/phylogeny.cgi). Based on this maximum likelihood tree, similar to previous finds by [23], Zip3 homologs can be divided into two groups: HEI10-like and Zip3/RNF212-like. This analysis shows that ZHP-4 is evolutionary more closely related to Zip/RNF212 members than to HEI10 members. (B) Protein alignment of the RING finger domain of *Sordaria macroscpora* (Sm) HEI10, *Mus musculus* (Mm) HEI10 and RNF212 and RNF4, *Homo sapiens* (Hs) HEI10 and RNF212, *Oryza sativa* (Os) HEI10, *Arabidopsis thaliana* (At) HEI10, *Caenorhabditis elegans* (Ce) ZHP-3 and ZHP-4, *Drosophila melanogaster* (Dm) Vilya, *Saccharomyces cerevisiae* (Sc) Zip3 and Slx8, and *Rattus norvegicus* Brca1 using MUSCLE (http://www.ebi.ac.uk/Tools/msa/muscle/). Conserved cysteines and histidines in the consensus sequence of the RING finger domain are in red, any residues that do not follow the consensus motif are in blue and underlined. Mutating conserved histidines (marked by black boxes) in Sc Zip3 [19] and Sm HEI10 [26] have been shown to result in meiotic phenotypes. Our study demonstrates that mutation of histidines in ZHP-4 but not ZHP-3 (marked in blue boxes) also results in chromosome nondisjunction.
(TIF)

S4 Fig ZHP-4 negatively regulate DSBs formation in TZ/early pachytene. (A) The numbers of RAD-51 foci were scored in each nucleus of the gonads of the indicated genotypes as reported for Fig 4. Young wild-type and *zhp-4(vv103)* mutant animals were injected with *rad-54* dsRNA, dissected two days post injection (approximately three days post L4), and stained with α-RAD-51 and α-HTP-3 antibodies. RAD-51 foci were scored only in zones 1–4 (corresponding to the mitotic region until early/mid-pachytene stages) because the effect of *rad-54(RNAi)* was not complete. In fact, the number of RAD-51 foci in our experiments does not accumulate (shown in part B) as reported previously [41]. The scored zones 1–4 demonstrate that the animals are affected by *rad-54(RNAi)* since RAD-51 numbers are significantly higher in zone 3 and 4 of injected animals versus wild types (*p*<0.001) and comparable to previously reported results [41]. Removing the ability to process RAD-51 foci in our null mutant results in a significant increase of foci in zone 4 in comparison to *rad-54(RNAi)* germlines (average of 13.5 foci/nucleus in *zhp-4(vv103);rad-54(RNAi)* versus 9.6 in *rad-54(RNAi)*, *p*<0.00001), supporting the conclusion that the elevated number of RAD-51 foci in our mutants is consistent with ZHP-4 being required to negatively regulate DSB formation rather than a manifesting a defect in their processing. Three gonads were analyzed for *zhp-4(vv103)* and two for wild types (Mann-Whitney test, *** *p*<0.001). (B) Immunostaining of RAD-51 (green) and the axis component HTP-3 (red) of injected animals dissected 3 days post injection (4 days post L4) show that the increased number of RAD-51 foci resulting from *rad-54(RNAi)* affects the germlines only until mid-pachytene since the foci start disappearing and are completely removed by the end of late pachytene. Scale bars, 10 μm.
(TIF)

S5 Fig ZHP-4^*vv96*^ fails to continuously localize along synapsed chromosomes. (A) Nuclei from early, mid and late pachytene of transgenic worms expressing *zhp-4*::*ha* tagged gene immunostained with anti-HA antibody (green) recapitulate the endogenous localization of ZHP-4. By mid-pachytene the HA tag is continuously associated with synapsed chromosomes and by late pachytene it is restricted to 6 foci/nucleus. (B) Representative images of early, mid and late pachytene nuclei of *zhp-4(vv96*::*ha)* germlines immunostained with anti-HA (green) and HTP-3 (red) antibodies showing punctate localization of ZHP-4::HA^*vv96*^ throughout pachytene stages. Scale bars, 5 μm.
(TIF)

S6 Fig *zhp-4* mutants are proficient in SMO-1 localization at pachytene stages. Representative images of late pachytene nuclei of indicated genotypes immunostained for SMO-1 in green (*C*. *elegans* SUMO homolog) and HTP-3 in red. SMO-1 is associated with chromatin in all genotypes with no detectable differences. Scale bars, 5 μm.
(TIF)

S7 Fig Aberrant spindle formation in *zhp-4(vv96)* mutants. (A) Partial projections of the oocyte metaphase I spindle in the indicated genotypes stained with DAPI (blue) and anti-tubulin (green) antibody. In wild-type oocytes, tubulin organizes about the congressed bivalents to form a bipolar spindle in which the tubulin channels are evident while in *zhp-4(vv96)*, both congression and spindle formation are disrupted. (B) Partial projections of the oocyte anaphase I spindle in the indicated genotypes. In wild-type oocytes, chromosomes segregate as two distinct chromatin masses towards opposite poles with the microtubule channels evident between them. An example of *zhp-4(vv96)* mutant nucleus at the same stage shows chromosomes masses that are still connected and appear tangled. Also the tubulin localization is disrupted and partially overlaps with the chromatin masses. (C) Additional examples of metaphase oocytes from *zhp-4(vv96)* mutants displayed a wide range of phenotypes: (left) chromosomes failed to align to the metaphase plate; (middle) chromosomes managed to align to the metaphase plate; (right) chromatin was not condensed despite proper spindle formation. Anaphase oocytes from *zhp-4(vv96)* animals show a defect in clean and neat chromosome segregation. Scale bars, 5 μm.
(TIF)

## References

[1] Heyer WD, Ehmsen KT, Liu J. Regulation of homologous recombination in eukaryotes. Annu Rev Genet. 2010;44:113–39. 10.1146/annurev-genet-051710-150955 20690856 10.1146/annurev-genet-051710-150955 PMC4114321

[2] de Massy B. Initiation of meiotic recombination: how and where? Conservation and specificities among eukaryotes. Annu Rev Genet. 2013;47: 563–599. 10.1146/annurev-genet-110711-155423 24050176 10.1146/annurev-genet-110711-155423

[3] Koehler KE, Hawley RS, Sherman S, Hassold T. Recombination and nondisjunction in humans and flies. Hum. Mol. Genet. 1996;5: 1495–1504. 8875256 10.1093/hmg/5.supplement_1.1495

[4] Mercier R, Mézard C, Jenczewski E, Macaisne N, Grelon M. The molecular biology of meiosis in plants. Annu Rev Plant Biol. 2015;66: 297–327). 10.1146/annurev-arplant-050213-035923 25494464 10.1146/annurev-arplant-050213-035923

[5] Hunter N. Meiotic Recombination: The Essence of Heredity. Cold Spring Harb Perspect Biol 7 2015. 10.1101/cshperspect.a016618 PMC4665078 26511629

[6] Nottke AC, Beese-Sims SE, Pantalena LF, Reinke V, Shi Y, Colaiácovo MP. SPR-5 is a histone H3K4 demethylase with a role in meiotic double-strand break repair. Proc Natl Acad Sci U S A. 2011;108(31):12805–10. 10.1073/pnas.1102298108 Epub 2011 Jul 18. 21768382 10.1073/pnas.1102298108 PMC3150895

[7] Rosu S, Libuda DE, Villeneuve AM. Robust crossover assurance and regulated interhomolog access maintain meiotic crossover number. Science. 2011;334(6060):1286–9. 10.1126/science.1212424 22144627 10.1126/science.1212424 PMC3360972

[8] Saito TT, Colaiácovo MP. Break to make a connection. PLoS Genet. 2011;7(2):e1002006 10.1371/journal.pgen.1002006 Epub 2011 Feb 24. 21383860 10.1371/journal.pgen.1002006 PMC3044676

[9] Schvarzstein M, Wignall SM, Villeneuve AM. Coordinating cohesion, co-orientation, and congression during meiosis: lessons from holocentric chromosomes. Genes Dev. 2010;24(3):219–28. 10.1101/gad.1863610 20123904 10.1101/gad.1863610 PMC2811823

[10] Barnes TM, Kohara Y, Coulson A, Hekimi S. Meiotic recombination, noncoding DNA and genomic organization in Caenorhabditis elegans. Genetics. 1995;141(1):159–79. 8536965 10.1093/genetics/141.1.159 PMC1206715

[11] Yokoo R, Zawadzki KA, Nabeshima K, Drake M, Arur S, Villeneuve AM. COSA-1 reveals robust homeostasis and separable licensing and reinforcement steps governing meiotic crossovers. Cell. 2012;149: 75–87. 10.1016/j.cell.2012.01.052 22464324 10.1016/j.cell.2012.01.052 PMC3339199

[12] Jagut M, Hamminger P, Woglar A, Millonigg S, Paulin L, Mikl M, et al Separable Roles for a Caenorhabditis elegans RMI1 Homolog in Promoting and Antagonizing Meiotic Crossovers Ensure Faithful Chromosome Inheritance. PLoS Biol. 2016;14: e1002412 10.1371/journal.pbio.1002412 27011106 10.1371/journal.pbio.1002412 PMC4807110

[13] Zalevsky J, MacQueen AJ, Duffy JB, Kemphues KJ, Villeneuve AM. Crossing over during Caenorhabditis elegans meiosis requires a conserved MutS-based pathway that is partially dispensable in budding yeast. Genetics. 1999;153(3):1271–83. 10545458 10.1093/genetics/153.3.1271 PMC1460811

[14] Kelly KO, Dernburg AF, Stanfield GM, Villeneuve AM. Caenorhabditis elegans msh-5 is required for both normal and radiation-induced meiotic crossing over but not for completion of meiosis. Genetics. 2000;156(2):617–30. 11014811 10.1093/genetics/156.2.617 PMC1461284

[15] Borner GV, Kleckner N, Hunter N. Crossover/noncrossover differentiation, synaptonemal complex formation, and regulatory surveillance at the leptotene/zygotene transition of meiosis. Cell. 2004;117: 29–45. 15066280 10.1016/s0092-8674(04)00292-2

[16] Rao HB, Qiao H, Bhatt SK, Bailey LR, Tran HD, Bourne SL, et al A SUMO-ubiquitin relay recruits proteasomes to chromosome axes to regulate meiotic recombination. Science. 2017;355: 403–407. 10.1126/science.aaf6407 28059716 10.1126/science.aaf6407 PMC5569317

[17] Ahuja JS, Sandhu R, Mainpal R, Lawson C, Henley H, Hunt PA, et al Control of meiotic pairing and recombination by chromosomally tethered 26S proteasome. Science. 2017;355: 408–411. 10.1126/science.aaf4778 28059715 10.1126/science.aaf4778 PMC6054871

[18] Agarwal S, Roeder GS. Zip3 provides a link between recombination enzymes and synaptonemal complex proteins. Cell. 2000;102: 245–255. 10943844 10.1016/s0092-8674(00)00029-5

[19] Cheng CH, Lo YH, Liang SS, Ti SC, Lin FM, Yeh CH, et al SUMO modifications control assembly of synaptonemal complex and polycomplex in meiosis of Saccharomyces cerevisiae. Genes Dev. 2006;20: 2067–2081. 10.1101/gad.1430406 16847351 10.1101/gad.1430406 PMC1536058

[20] Toby GG, Gherraby W, Coleman TR, Golemis EA. A novel RING finger protein, human enhancer of invasion 10, alters mitotic progression through regulation of cyclin B levels. Mol Cell Biol. 2003;23: 2109–2122. 10.1128/MCB.23.6.2109-2122.2003 12612082 10.1128/MCB.23.6.2109-2122.2003 PMC149478

[21] Jantsch V, Pasierbek P, Mueller MM, Schweizer D, Jantsch M, Loidl J. Targeted gene knockout reveals a role in meiotic recombination for ZHP-3, a Zip3-related protein in Caenorhabditis elegans. Mol Cell Biol. 2004;24: 7998–8006. 10.1128/MCB.24.18.7998-8006.2004 15340062 10.1128/MCB.24.18.7998-8006.2004 PMC515049

[22] Bhalla N, Wynne DJ, Jantsch V, Dernburg AF. ZHP-3 acts at crossovers to couple meiotic recombination with synaptonemal complex disassembly and bivalent formation in C. elegans. PLoS Genet. 2008;4: e1000235 10.1371/journal.pgen.1000235 18949042 10.1371/journal.pgen.1000235 PMC2567099

[23] Chelysheva L, Vezon D, Chambon A, Gendrot G, Pereira L, Lemhemdi A, et al The Arabidopsis HEI10 is a new ZMM protein related to Zip3. PLoS Genet. 2012;8: e1002799 10.1371/journal.pgen.1002799 22844245 10.1371/journal.pgen.1002799 PMC3405992

[24] Wang K, Wang M, Tang D, Shen Y, Miao C, Hu Q, et al The role of rice HEI10 in the formation of meiotic crossovers. PLoS Genet. 2012;8: e1002809 10.1371/journal.pgen.1002809 22792078 10.1371/journal.pgen.1002809 PMC3390396

[25] Reynolds A, Qiao H, Yang Y, Chen JK, Jackson N, Biswas K, et al RNF212 is a dosage-sensitive regulator of crossing-over during mammalian meiosis. Nat Genet. 2013;45: 269–278. 10.1038/ng.2541 23396135 10.1038/ng.2541 PMC4245152

[26] De Muyt A, Zhang L, Piolot T, Kleckner N, Espagne E, Zickler D. E3 ligase Hei10: a multifaceted structure-based signaling molecule with roles within and beyond meiosis. Genes Dev. 2014;28: 1111–1123. 10.1101/gad.240408.114 24831702 10.1101/gad.240408.114 PMC4035539

[27] Qiao H, Prasada Rao HB, Yang Y, Fong JH, Cloutier JM, Deacon DC, et al Antagonistic roles of ubiquitin ligase HEI10 and SUMO ligase RNF212 regulate meiotic recombination. Nat Genet. 2014;46: 194–199. 10.1038/ng.2858 24390283 10.1038/ng.2858 PMC4356240

[28] Lake CM, Nielsen RJ, Guo F, Unruh JR, Slaughter BD, Hawley RS. Vilya, a component of the recombination nodule, is required for meiotic double-strand break formation in Drosophila. Elife. 2015;4: e08287 10.7554/eLife.08287 26452093 10.7554/eLife.08287 PMC4703084

[29] Zhang L, Köhler S, Rillo-Bohn R, Dernburg AF. A compartmentalized signaling network mediates crossover control in meiosis. Elife. 2018;7 pii: e30789. 10.7554/eLife.30789 29521627 10.7554/eLife.30789 PMC5906097

[30] Janssens FA. The chiasmatype theory. A new interpretation of the maturation divisions. Cellule. 1909;25: 389–411. 10.1534/genetics.112.139725 PMC3374304 22701051

[31] Tease C, Jones GH. Do chiasmata disappear? An examination of whether closely spaced chiasmata are liable to reduction or loss. Chromosome Res. 1995;3: 162–168. 7780659 10.1007/BF00710709

[32] Nabeshima K, Villeneuve AM, Colaiácovo MP. Crossing over is coupled to late meiotic prophase bivalent differentiation through asymmetric disassembly of the SC. J Cell Biol. 2005;168(5):683–9. 10.1083/jcb.200410144 15738262 10.1083/jcb.200410144 PMC2171829

[33] Martinez-Perez E, Schvarzstein M, Barroso C, Lightfoot J, Dernburg AF, Villeneuve AM. Crossovers trigger a remodeling of meiotic chromosome axis composition that is linked to two-step loss of sister chromatid cohesion. Genes Dev. 2008;22(20):2886–901. 10.1101/gad.1694108 18923085 10.1101/gad.1694108 PMC2569886

[34] Woglar A, Villeneuve AM. Dynamic Architecture of DNA Repair Complexes and the Synaptonemal Complex at Sites of Meiotic Recombination. Cell. 2018 pii: S0092-8674(18)30398-2. 10.1016/j.cell.2018.03.066 [Epub ahead of print] 29754818 10.1016/j.cell.2018.03.066 PMC6003859

[35] Gareau JR, Lima CD. The SUMO pathway: emerging mechanisms that shape specificity, conjugation and recognition. Nat Rev Mol Cell Biol. 2010;11: 861–871. 10.1038/nrm3011 21102611 10.1038/nrm3011 PMC3079294

[36] Hodgkin J, Horvitz HR, Brenner S. Nondisjunction Mutants of the Nematode CAENORHABDITIS ELEGANS. Genetics. 1979;91(1):67–94. 17248881 10.1093/genetics/91.1.67 PMC1213932

[37] Alpi A, Pasierbek P, Gartner A, Loidl J. Genetic and cytological characterization of the recombination protein RAD-51 in Caenorhabditis elegans. Chromosoma. 2003;112(1):6–16. Epub 2003 Apr 8. 10.1007/s00412-003-0237-5 12684824 10.1007/s00412-003-0237-5

[38] Colaiácovo MP, MacQueen AJ, Martinez-Perez E, McDonald K, Adamo A, La Volpe A, et al Synaptonemal complex assembly in C. elegans is dispensable for loading strand-exchange proteins but critical for proper completion of recombination. Dev Cell. 2003;5(3):463–74. 12967565 10.1016/s1534-5807(03)00232-6

[39] Dernburg AF, McDonald K, Moulder G, Barstead R, Dresser M, Villeneuve AM. Meiotic recombination in C. elegans initiates by a conserved mechanism and is dispensable for homologous chromosome synapsis. Cell. 1998;94(3):387–98. 9708740 10.1016/s0092-8674(00)81481-6

[40] Metzger MB, Pruneda JN, Klevit RE, Weissman AM. RING-type E3 ligases: master manipulators of E2 ubiquitin-conjugating enzymes and ubiquitination. Biochim Biophys Acta. 2014;1843(1):47–60. 10.1016/j.bbamcr.2013.05.026 Epub 2013 Jun 6. 23747565 10.1016/j.bbamcr.2013.05.026 PMC4109693

[41] Mets DG, Meyer BJ. Condensins regulate meiotic DNA break distribution, thus crossover frequency, by controlling chromosome structure. Cell. 2009;139(1):73–86. 10.1016/j.cell.2009.07.035 Epub 2009 Sep 24. 19781752 10.1016/j.cell.2009.07.035 PMC2785808

[42] Yu Y, Ren JY, Zhang JM, Suo F, Fang XF, Wu F, et al A proteome-wide visual screen identifies fission yeast proteins localizing to DNA double-strand breaks. DNA Repair (Amst). 2013;12(6):433–43. 10.1016/j.dnarep.2013.04.001 23628481 10.1016/j.dnarep.2013.04.001

[43] Tang S, Wu MK, Zhang R, Hunter N. Pervasive and essential roles of the Top3-Rmi1 decatenase orchestrate recombination and facilitate chromosome segregation in meiosis. Mol Cell. 2015;57: 607–621 10.1016/j.molcel.2015.01.021 25699709 10.1016/j.molcel.2015.01.021 PMC4791043

[44] O'Neil NJ, Martin JS, Youds JL, Ward JD, Petalcorin MI, Rose AM, et al Joint molecule resolution requires the redundant activities of MUS-81 and XPF-1 during Caenorhabditis elegans meiosis. PLoS Genet. 2013;9(7):e1003582 10.1371/journal.pgen.1003582 Epub 2013 Jul 18. 23874209 10.1371/journal.pgen.1003582 PMC3715453

[45] Agostinho A, Meier B, Sonneville R, Jagut M, Woglar A, Blow J, et al Combinatorial regulation of meiotic holliday junction resolution in C. elegans by HIM-6 (BLM) helicase, SLX-4, and the SLX-1, MUS-81 and XPF-1 nucleases. PLoS Genet. 2013;9(7):e1003591 10.1371/journal.pgen.1003591 23901331 10.1371/journal.pgen.1003591 PMC3715425

[46] Kaitna S, Pasierbek P, Jantsch M, Loidl J, Glotzer M. The aurora B kinase AIR-2 regulates kinetochores during mitosis and is required for separation of homologous Chromosomes during meiosis. Curr Biol. 2002;12: 798–812. 12015116 10.1016/s0960-9822(02)00820-5

[47] Rogers E, Bishop JD, Waddle JA, Schumacher JM, Lin R. The aurora kinase AIR-2 functions in the release of chromosome cohesion in Caenorhabditis elegans meiosis. J Cell Biol. 2002;157: 219–229. 10.1083/jcb.200110045 11940606 10.1083/jcb.200110045 PMC1855215

[48] Pelisch F, Tammsalu T, Wang B, Jaffray EG, Gartner A, Hay RT. A SUMO-Dependent Protein Network Regulates Chromosome Congression during Oocyte Meiosis. Mol Cell. 2017;65(1):66–77. 10.1016/j.molcel.2016.11.001 Epub 2016 Dec 8. 27939944 10.1016/j.molcel.2016.11.001 PMC5222697

[49] Wignall SM, Villeneuve AM. Lateral microtubule bundles promote chromosome alignment during acentrosomal oocyte meiosis. Nat Cell Biol. 2009;11: 839–844. 10.1038/ncb1891 19525937 10.1038/ncb1891 PMC2760407

[50] Muscat CC, Torre-Santiago KM, Tran MV, Powers JA, Wignall SM. Kinetochore-independent chromosome segregation driven by lateral microtubule bundles. Elife. 2015;4: e06462 10.7554/eLife.06462 26026148 10.7554/eLife.06462 PMC4481507

[51] Joukov V, Chen J, Fox EA, Green JB, Livingston DM. Functional communication between endogenous BRCA1 and its partner, BARD1, during Xenopus laevis development. Proc Natl Acad Sci U S A. 2001;98: 12078–12083. 10.1073/pnas.211427098 11593018 10.1073/pnas.211427098 PMC59770

[52] Brzovic PS, Rajagopal P, Hoyt DW, King MC, Klevit RE. Structure of a BRCA1-BARD1 heterodimeric RING-RING complex. Nat Struct Biol. 2001;8: 833–837. 10.1038/nsb1001-833 11573085 10.1038/nsb1001-833

[53] Christensen DE, Brzovic PS, Klevit RE. E2-BRCA1 RING interactions dictate synthesis of mono- or specific polyubiquitin chain linkages. Nat Struct Mol Biol. 2007;14: 941–948. 10.1038/nsmb1295 17873885 10.1038/nsmb1295

[54] Wang Z, Jones GM, Prelich G. Genetic analysis connects SLX5 and SLX8 to the SUMO pathway in Saccharomyces cerevisiae. Genetics. 2006;172: 1499–1509. 10.1534/genetics.105.052811 16387868 10.1534/genetics.105.052811 PMC1456262

[55] Yang L, Mullen JR, Brill SJ. Purification of the yeast Slx5-Slx8 protein complex and characterization of its DNA-binding activity. Nucleic Acids Res. 2006;34: 5541–5551. 10.1093/nar/gkl685 17020915 10.1093/nar/gkl685 PMC1635298

[56] Uzunova K, Gottsche K, Miteva M, Weisshaar SR, Glanemann C, Schnellhardt M, et al Ubiquitin-dependent proteolytic control of SUMO conjugates. J Biol Chem. 2007;282: 34167–34175. 10.1074/jbc.M706505200 17728242 10.1074/jbc.M706505200

[57] Xie Y, Kerscher O, Kroetz MB, McConchie HF, Sung P, Hochstrasser M. The yeast Hex3.Slx8 heterodimer is a ubiquitin ligase stimulated by substrate sumoylation. J Biol Chem. 2007;282: 34176–34184. 10.1074/jbc.M706025200 17848550 10.1074/jbc.M706025200

[58] Reichman R, Shi Z, Malone R, Smolikove S. Mitotic and Meiotic Functions for the SUMOylation Pathway in the Caenorhabditis elegans Germline. Genetics. 2018;208(4):1421–1441. 10.1534/genetics.118.300787 Epub 2018 Feb 22. 29472245 10.1534/genetics.118.300787 PMC5887140

[59] Shinohara M, Oh SD, Hunter N, Shinohara A. Crossover assurance and crossover interference are distinctly regulated by the ZMM proteins during yeast meiosis. Nat Genetics. 2008;40: 299–309. 10.1038/ng.83 18297071 10.1038/ng.83

[60] Zetka M, Rose AM. Mutant rec-1 eliminates the meiotic pattern of crossing over in Caenorhabditis elegans. Genetics. 1995;141: 1339–1349. 8601478 10.1093/genetics/141.4.1339 PMC1206871

[61] MacQueen AJ, Colaiácovo MP, McDonald K, Villeneuve AM. Synapsis-dependent and -independent mechanisms stabilize homolog pairing during meiotic prophase in C. elegans. Genes Dev. 2002;16(18):2428–42. 10.1101/gad.1011602 12231631 10.1101/gad.1011602 PMC187442

[62] Zickler D, Kleckner N. The leptotene-zygotene transition of meiosis. Annu Rev Genet. 1998;32:619–97. 10.1146/annurev.genet.32.1.619 9928494 10.1146/annurev.genet.32.1.619

[63] Brenner S. The genetics of Caenorhabditis elegans. Genetics. 1974;77(1):71–94. 4366476 10.1093/genetics/77.1.71 PMC1213120

[64] Paix A, Folkmann A, Rasoloson D, Seydoux G. High Efficiency, Homology-Directed Genome Editing in Caenorhabditis elegans Using CRISPR-Cas9 Ribonucleoprotein Complexes. Genetics. 2015;201(1):47–54. 10.1534/genetics.115.179382 Epub 2015 Jul 17. 26187122 10.1534/genetics.115.179382 PMC4566275

[65] Fire A, Xu S, Montgomery MK, Kostas SA, Driver SE, Mello CC. Potent and specific genetic interference by double-stranded RNA in Caenorhabditis elegans. Nature. 1998;391(6669):806–11. 10.1038/35888 9486653 10.1038/35888

