# Supplementary material for: C. elegans ZHP-4 is required at multiple distinct steps in the formation of crossovers and their transition to segregation competent chiasmata
Source: PLoS Genet. 2018 Oct 31;14(10):e1007776. doi: 10.1371/journal.pgen.1007776 (PMC6239344; doi:10.1371/journal.pgen.1007776)
Supplement: S1 Table — (DOCX) [file pgen.1007776.s001.docx]

Nguyen_TabS1

| **Mutation** | **sgRNA** | **Repair template (mutations are in lower case)** | **Forward primer** | **Reverse primer** | **Restriction enzyme digest** |
| --- | --- | --- | --- | --- | --- |
| *zhp-4(vv103)* | TTCAATGGTTCTTCTTCCGG | None | CATTTTTCAGCCAAAAACTCA | TCGTTCTCGAATTTTCCCATA | BspEI  WT: 833  Mutant: 599 and 234 |
| *zhp-4(H26A)* | TTCAATGGTTCTTCTTCCGG | CCGGTGTTACAAATTTCCGTCAAAACAAATCGAATTCTATCTGACAAATTGTATGgccATGTTCTGTATTGAATGTGAACGATTATGTCATCCACCGGAgGAAGAACCATTGAA | CATTTTTCAGCCAAAAACTCA | TCGTTCTCGAATTTTCCCATA | NdeI  WT: 636 and 202 bp  Mutant: 838 bp |
| *zhp4(vv96::ha)* | GAACGGGGCGGAGTCTGAGG | CACGAAATTTCGAAACTAAAAAAGCAACTGGCCGAGCAGGCTCCGCCCCCTtacccatatgatgtcccggattacgcttagACTCCGCCCCGTTCAAATTCTCTGAAAGTGGCGA | TTCGATCGACTACGTGATGC | GTAATCCGGGACATCATATGG | Mutant: positive PCR product |
| *zhp-3(H25A)* | TTCAACCGAAAACCACCGGA | CACTGTAATAAATGCTTCAACCGAAAACCACCaGATGGATTCTTCATCTCTTCATGTTTTgccATTTTTTGTACGAAATGCGCGAAAGCAGGTACGAAAATTAGTTTAAAATCTTGAAAA | CCGAGCTGCTAAACGACATT | GCAAGACTGAATGGAAATGGA | HpaII  WT: 641 and 151 bp  Mutant: 792 bp |
