# Supplementary figures and images for: C. elegans ZHP-4 is required at multiple distinct steps in the formation of crossovers and their transition to segregation competent chiasmata
Source: PLoS Genet. 2018 Oct 31;14(10):e1007776. doi: 10.1371/journal.pgen.1007776 (PMC6239344; doi:10.1371/journal.pgen.1007776)

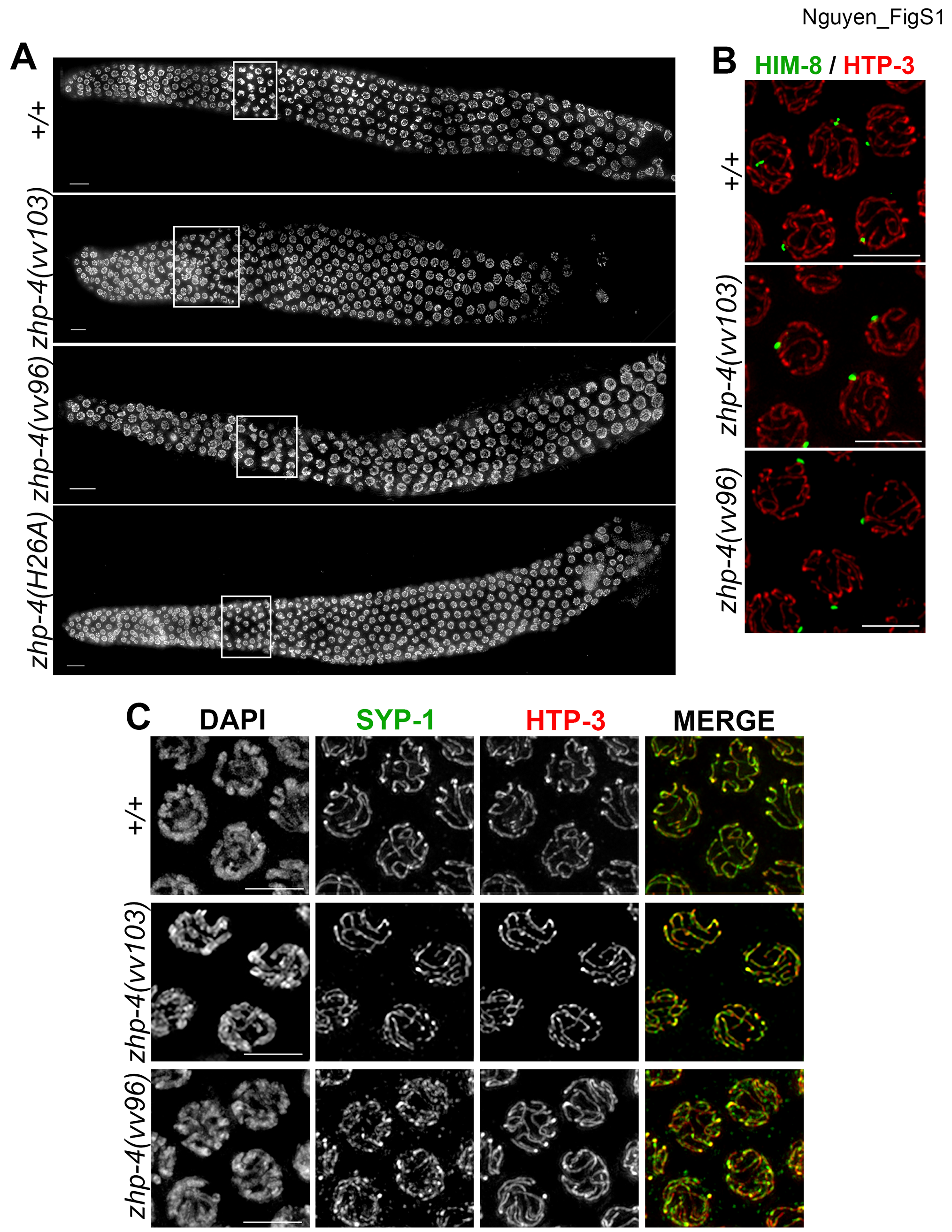

Supplement: S1 Fig — (A) Representative gonads from wild-type and zhp-4 mutant animals show that nuclear morphology as assessed by DAPI staining is not affected in the mutants. Transition zone of each gonad is marked by the white boxes. Scale bars 10μm. (B) Immunostaining for HIM-8 in green (X chromosome pairing center binding protein) show the X chromosomes to be paired in all mid-pachytene nuclei of both zhp-4 mutants. (C) Representative images of mid-pachytene nuclei immunostained for SYP-1 in green (transverse element of the SC) and HTP-3 in red (axial element of the SC) on wild-type and zhp-4 germlines showing no defect in SC assembly. Scale bars 5μm. (TIF) [file pgen.1007776.s002.tif]

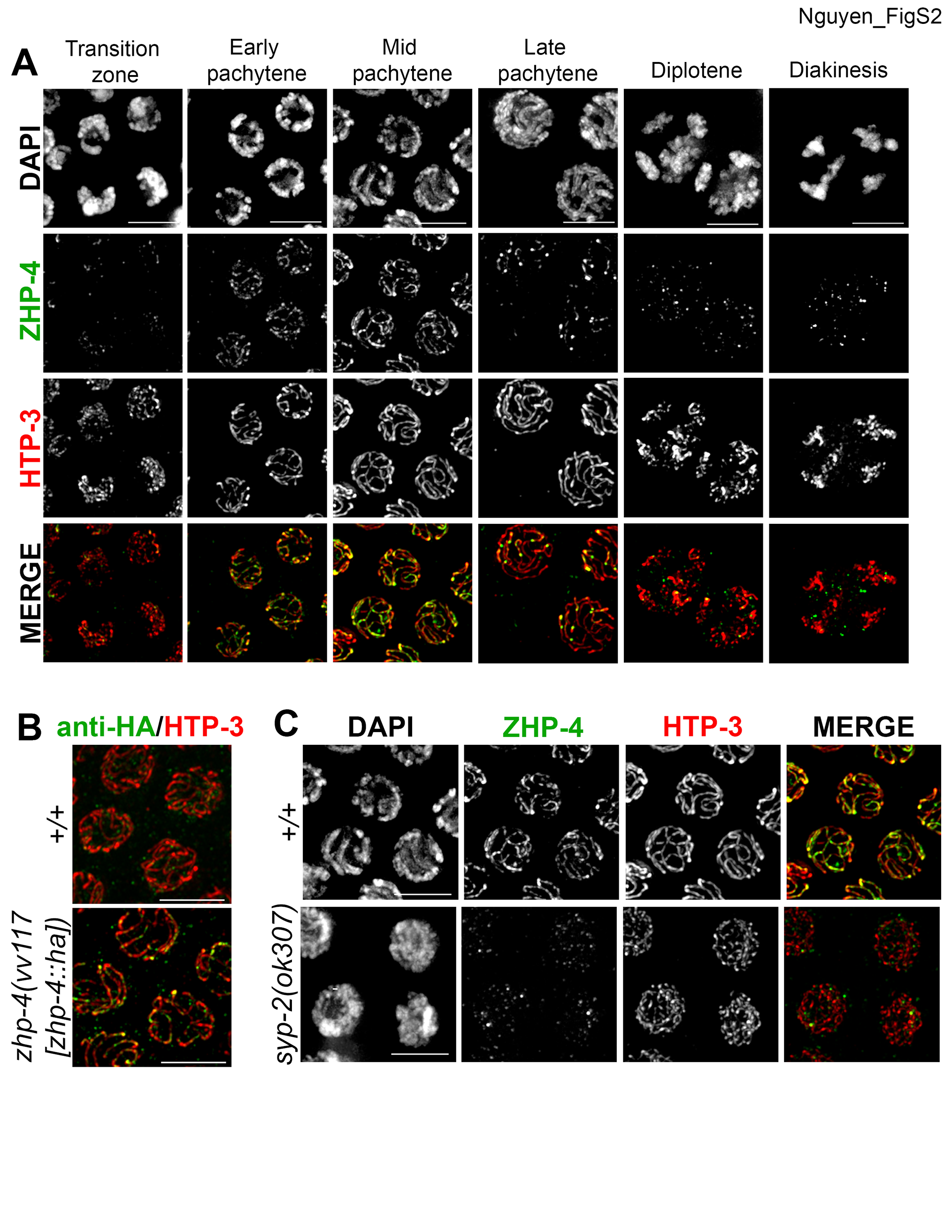

Supplement: S2 Fig — (A) Representative images of different stages of meiotic prophase I in wild-type gonads immunostained for ZHP-4 (green) and HTP-3 (red) antibodies. ZHP-4 is first seen as bright foci at transition zone. Starting from early pachytene, it can be detected along the chromosome tracks. Transitioning from mid to late pachytene, ZHP-4 is restricted to shorter stretches and eventually to six foci per nucleus on average, occasionally shown as short stretches at late pachytene. Some of these foci can be observed at diplotene but they were completely removed by early diakinesis. (B) Representative images of mid-pachytene nuclei of both wild-type and zhp-4::ha germlines immunostained with anti-HA (green) and HTP-3 (red) antibodies. No specific signal is detected in wild-type germlines, whereas linear tracks of ZHP-4::HA colocalize with HTP-3 in zhp-4::ha germlines. (C) Immunostaining for ZHP-4 (green) and HTP-3 (red) in wild-type and syp-2(ok307) germlines showed that the recruitment of ZHP-4 to meiotic chromosomes is dependent on the SC proteins. Scale bars 5μm. (TIF) [file pgen.1007776.s003.tif]

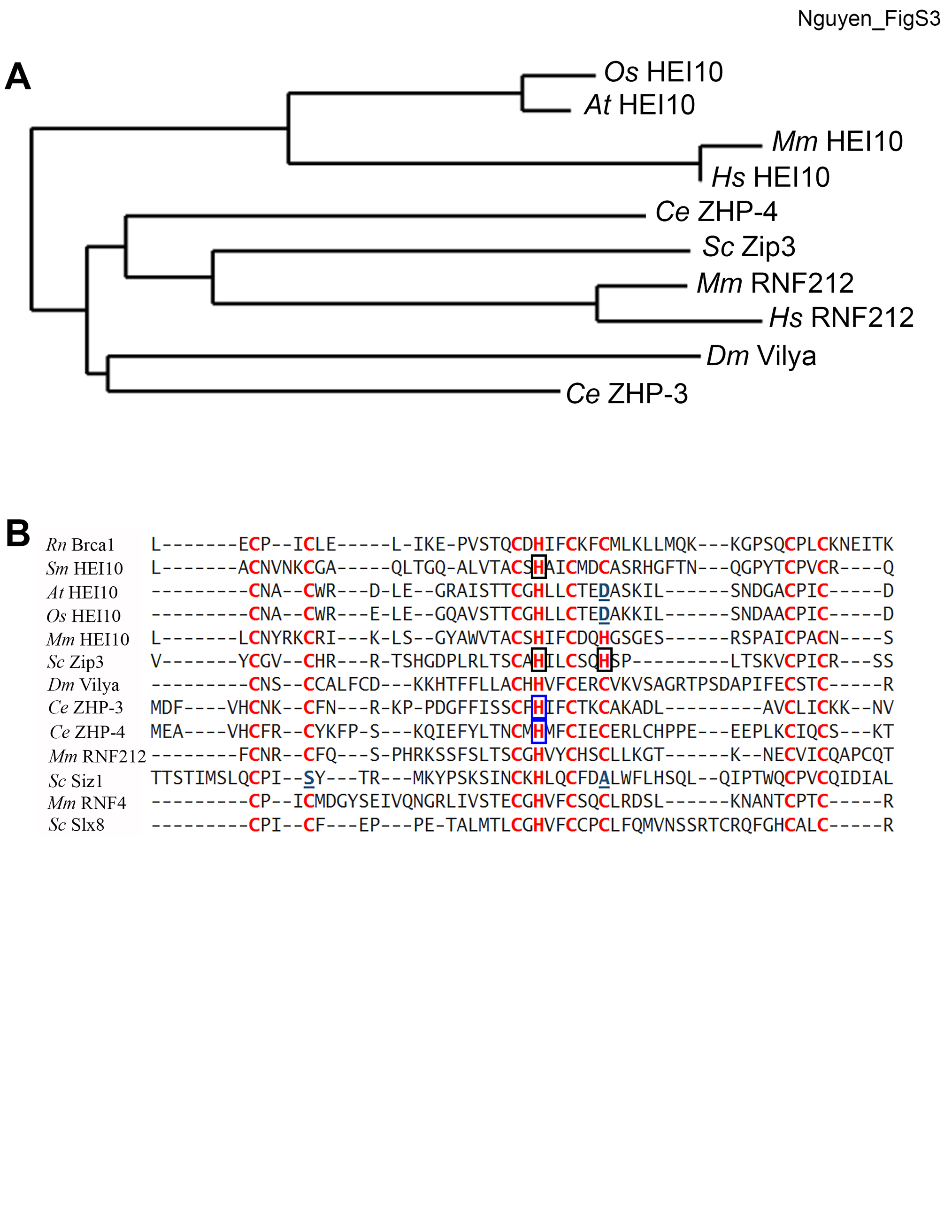

Supplement: S3 Fig — (A) Maximum likelihood tree constructed from a multiple whole protein sequence alignment of Mus musculus (Mm) HEI10 and RNF212, Homo sapiens (Hs) HEI10 and RNF212, Oryza sativa (Os) HEI10, Arabidopsis thaliana (At) HEI10, Caenorhabditis elegans (Ce) ZHP-3 and ZHP-4, Drosophila melanogaster (Dm) Vilya and Saccharomyces cerevisiae (Sc) Zip3. Proteins were aligned using MUSCLE and a phylogenetic maximum likelihood tree was constructed using Phylogeny Analysis (http://www.phylogeny.fr/phylogeny.cgi). Based on this maximum likelihood tree, similar to previous finds by [23], Zip3 homologs can be divided into two groups: HEI10-like and Zip3/RNF212-like. This analysis shows that ZHP-4 is evolutionary more closely related to Zip/RNF212 members than to HEI10 members. (B) Protein alignment of the RING finger domain of Sordaria macroscpora (Sm) HEI10, Mus musculus (Mm) HEI10 and RNF212 and RNF4, Homo sapiens (Hs) HEI10 and RNF212, Oryza sativa (Os) HEI10, Arabidopsis thaliana (At) HEI10, Caenorhabditis elegans (Ce) ZHP-3 and ZHP-4, Drosophila melanogaster (Dm) Vilya, Saccharomyces cerevisiae (Sc) Zip3 and Slx8, and Rattus norvegicus Brca1 using MUSCLE (http://www.ebi.ac.uk/Tools/msa/muscle/). Conserved cysteines and histidines in the consensus sequence of the RING finger domain are in red, any residues that do not follow the consensus motif are in blue and underlined. Mutating conserved histidines (marked by black boxes) in Sc Zip3 [19] and Sm HEI10 [26] have been shown to result in meiotic phenotypes. Our study demonstrates that mutation of histidines in ZHP-4 but not ZHP-3 (marked in blue boxes) also results in chromosome nondisjunction. (TIF) [file pgen.1007776.s004.tif]

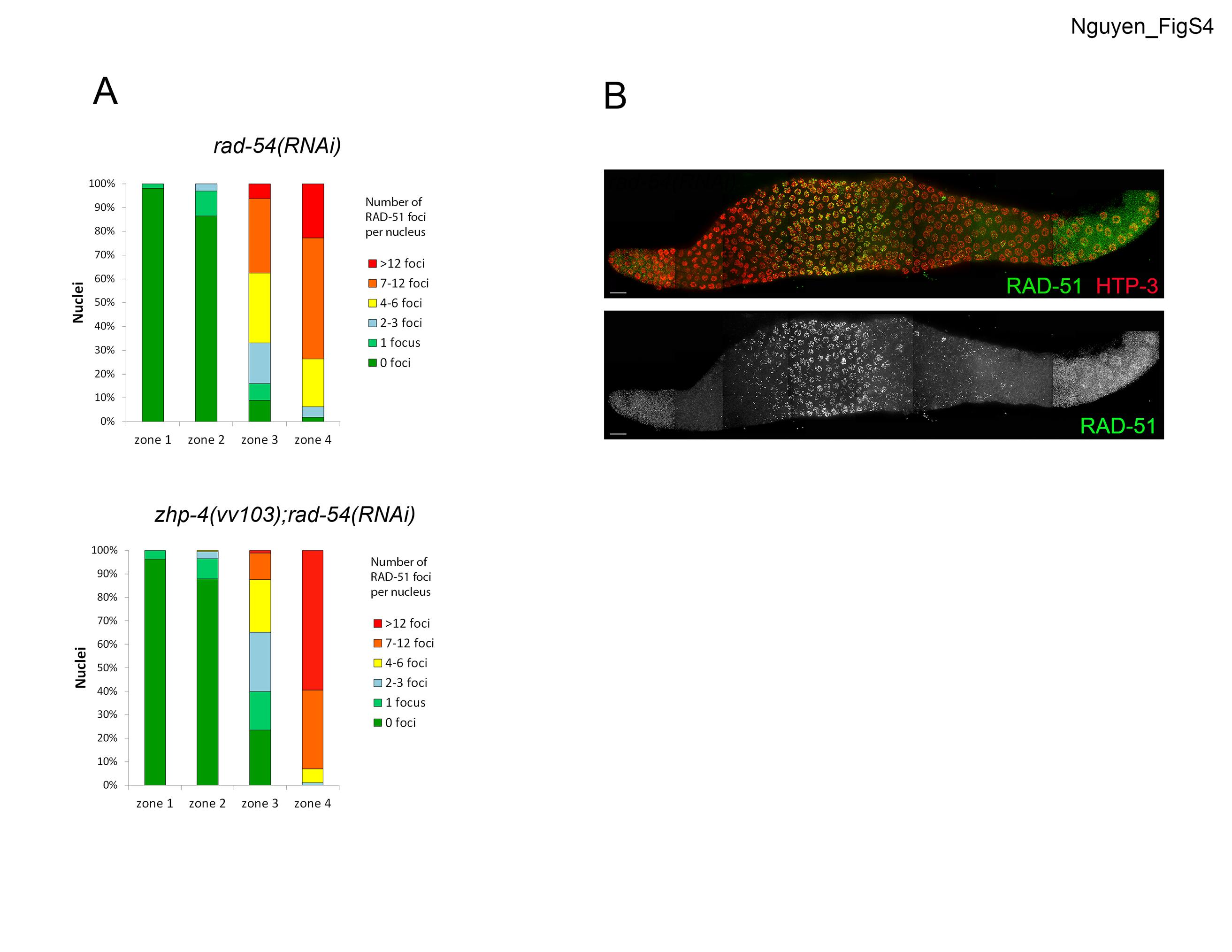

Supplement: S4 Fig — (A) The numbers of RAD-51 foci were scored in each nucleus of the gonads of the indicated genotypes as reported for Fig 4. Young wild-type and zhp-4(vv103) mutant animals were injected with rad-54 dsRNA, dissected two days post injection (approximately three days post L4), and stained with α-RAD-51 and α-HTP-3 antibodies. RAD-51 foci were scored only in zones 1–4 (corresponding to the mitotic region until early/mid-pachytene stages) because the effect of rad-54(RNAi) was not complete. In fact, the number of RAD-51 foci in our experiments does not accumulate (shown in part B) as reported previously [41]. The scored zones 1–4 demonstrate that the animals are affected by rad-54(RNAi) since RAD-51 numbers are significantly higher in zone 3 and 4 of injected animals versus wild types (p<0.001) and comparable to previously reported results [41]. Removing the ability to process RAD-51 foci in our null mutant results in a significant increase of foci in zone 4 in comparison to rad-54(RNAi) germlines (average of 13.5 foci/nucleus in zhp-4(vv103);rad-54(RNAi) versus 9.6 in rad-54(RNAi), p<0.00001), supporting the conclusion that the elevated number of RAD-51 foci in our mutants is consistent with ZHP-4 being required to negatively regulate DSB formation rather than a manifesting a defect in their processing. Three gonads were analyzed for zhp-4(vv103) and two for wild types (Mann-Whitney test, *** p<0.001). (B) Immunostaining of RAD-51 (green) and the axis component HTP-3 (red) of injected animals dissected 3 days post injection (4 days post L4) show that the increased number of RAD-51 foci resulting from rad-54(RNAi) affects the germlines only until mid-pachytene since the foci start disappearing and are completely removed by the end of late pachytene. Scale bars, 10 μm. (TIF) [file pgen.1007776.s005.tif]

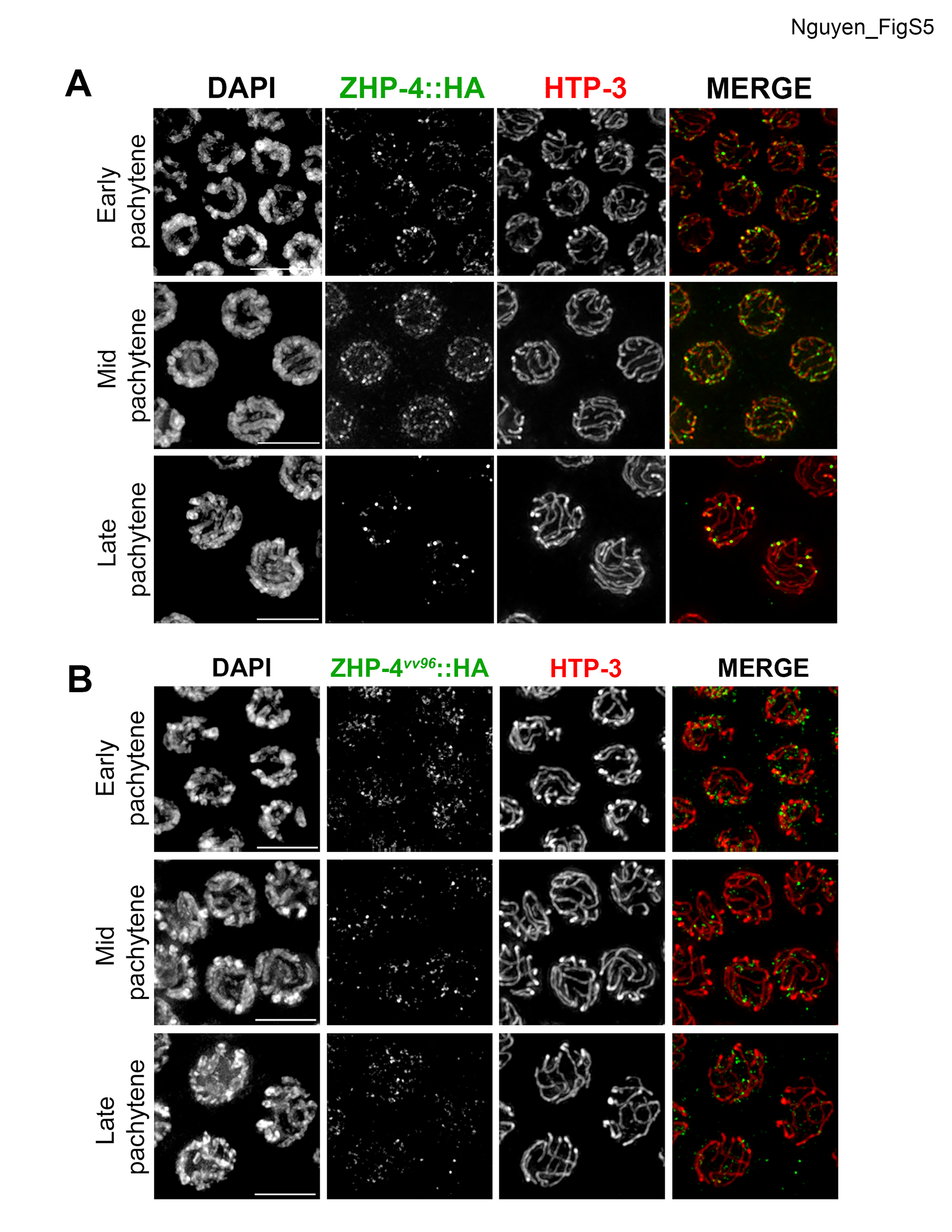

Supplement: S5 Fig — (A) Nuclei from early, mid and late pachytene of transgenic worms expressing zhp-4::ha tagged gene immunostained with anti-HA antibody (green) recapitulate the endogenous localization of ZHP-4. By mid-pachytene the HA tag is continuously associated with synapsed chromosomes and by late pachytene it is restricted to 6 foci/nucleus. (B) Representative images of early, mid and late pachytene nuclei of zhp-4(vv96::ha) germlines immunostained with anti-HA (green) and HTP-3 (red) antibodies showing punctate localization of ZHP-4::HAvv96 throughout pachytene stages. Scale bars, 5 μm. (TIF) [file pgen.1007776.s006.tif]

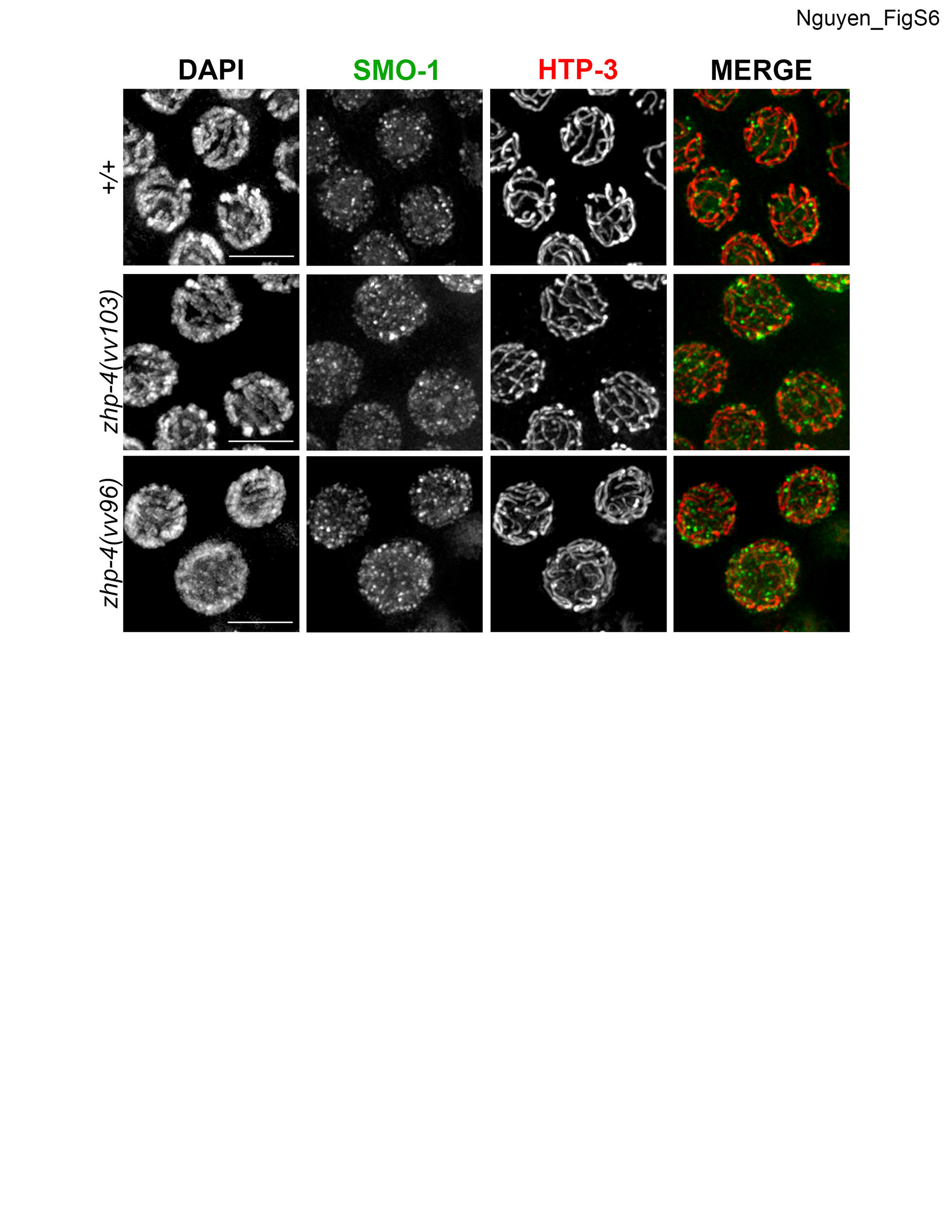

Supplement: S6 Fig — Representative images of late pachytene nuclei of indicated genotypes immunostained for SMO-1 in green (C. elegans SUMO homolog) and HTP-3 in red. SMO-1 is associated with chromatin in all genotypes with no detectable differences. Scale bars, 5 μm. (TIF) [file pgen.1007776.s007.tif]

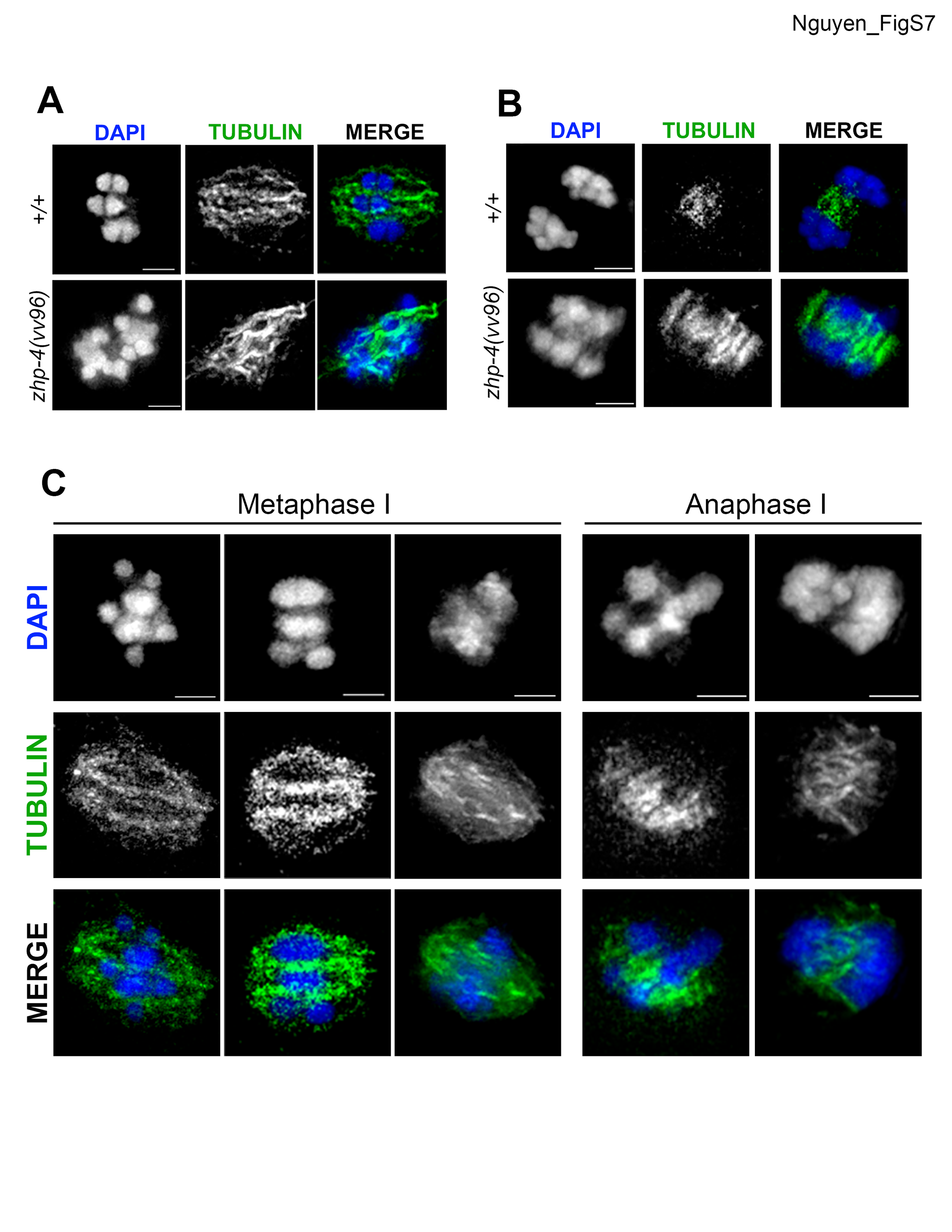

Supplement: S7 Fig — (A) Partial projections of the oocyte metaphase I spindle in the indicated genotypes stained with DAPI (blue) and anti-tubulin (green) antibody. In wild-type oocytes, tubulin organizes about the congressed bivalents to form a bipolar spindle in which the tubulin channels are evident while in zhp-4(vv96), both congression and spindle formation are disrupted. (B) Partial projections of the oocyte anaphase I spindle in the indicated genotypes. In wild-type oocytes, chromosomes segregate as two distinct chromatin masses towards opposite poles with the microtubule channels evident between them. An example of zhp-4(vv96) mutant nucleus at the same stage shows chromosomes masses that are still connected and appear tangled. Also the tubulin localization is disrupted and partially overlaps with the chromatin masses. (C) Additional examples of metaphase oocytes from zhp-4(vv96) mutants displayed a wide range of phenotypes: (left) chromosomes failed to align to the metaphase plate; (middle) chromosomes managed to align to the metaphase plate; (right) chromatin was not condensed despite proper spindle formation. Anaphase oocytes from zhp-4(vv96) animals show a defect in clean and neat chromosome segregation. Scale bars, 5 μm. (TIF) [file pgen.1007776.s008.tif]
